# Association of carotid atherosclerosis with brain tissue integrity and metabolic parameters in type 2 diabetes patients

**DOI:** 10.3389/fendo.2025.1586085

**Published:** 2025-08-01

**Authors:** Seung Hoon Lim, Chang-Woo Ryu, Yunan Tian, Ji Eun Jun, Soonchan Park, In-Kyung Jeong, Geon-Ho Jahng

**Affiliations:** ^1^ Department of Neurosurgery, Kyung Hee University Hospital at Gangdong, Kyung Hee University College of Medicine, Seoul, Republic of Korea; ^2^ Department of Radiology, Kyung Hee University Hospital at Gangdong, Kyung Hee University College of Medicine, Seoul, Republic of Korea; ^3^ Department of Medicine, Graduate School, Kyung Hee University College of Medicine, Seoul, Republic of Korea; ^4^ Department of Endocrinology and Metabolism, Kyung Hee University Hospital at Gangdong, Kyung Hee University College of Medicine., Seoul, Republic of Korea

**Keywords:** type 2 diabetes, brain, carotid artery plaque, gray-white matter junction volume, metabolic parameters

## Abstract

**Background:**

Type 2 diabetes mellitus (T2DM) is known to adversely impact brain health, leading to cognitive decline and brain tissue volume reduction. This study aimed to assess the damage to gray-white matter junction tissue volume (gwJTV) in T2DM patients with and without carotid artery plaques, and its association with various metabolic parameters.

**Methods:**

We conducted a cross-sectional study involving 69 T2DM patients, employing three-dimensional T1-weighted MRI scans to measure brain tissue volumes, particularly gwJTV, and analyzing blood samples for metabolic parameters. Voxel-based (VBA) and region-of-interest (ROI) analyses of gwJTV were performed to evaluate the group difference with and without carotid artery plaques and to determine correlations to metabolic biomarkers.

**Results:**

Voxel-based and region-of-interest analyses revealed that participants with carotid plaques had lower gwJTV than those without at the specific brain area. ROI results study further demonstrated positive associations between gwJTV and metabolic parameters such as AST, ApoB, and LDL, and negative associations with C-peptide, creatinine, and hsCRP.

**Conclusion:**

Our findings suggest that gwJTV could be a valuable imaging biomarker for monitoring brain and vascular health in T2DM patients, particularly those affected by carotid atherosclerosis.

## Highlights

- Gray-white matter junction tissue volume (gwJTV) was significantly lower in T2DM patients with carotid plaques compared to those without, indicating the detrimental impact of carotid atherosclerosis on brain tissue integrity.
- The study found that gwJTV was positively associated with metabolic biomarkers such as AST, ApoB, LDL, and others, while negatively associated with C-peptide, creatinine, and hsCRP, highlighting complex metabolic interactions affecting brain health.
- gwJTV might serve as a potential imaging biomarker for monitoring both brain and vascular health in T2DM patients, especially those with carotid atherosclerosis.

## 1 Introduction

Type 2 diabetes mellitus (T2DM), characterized by insulin resistance and relative insulin deficiency, leads not only to chronic hyperglycemia but also to dyslipidemia and various metabolic abnormalities, resulting in complications across multiple organs. Traditionally, diabetic complications were categorized into microvascular complications, affecting the retina, kidneys, and nerves, and macrovascular complications, caused by atherosclerosis in the cerebral, coronary, and peripheral arteries. However, recent classifications have redefined these complications into three pathological categories: vascular, parenchymal, and hybrid (vascular plus parenchymal) (1). Diabetes-induced tissue damage originates in the vasculature due to hyperglycemia and dyslipidemia. However, it is well established that in the myocardium and central nervous system (CNS), not only vascular dysfunction but also the impairment of surrounding parenchymal cells is involved. Hyperglycemia over time can damage blood vessels in the brain, leading to reduced blood flow and brain cell death.

Neuroimaging studies have shown that T2DM patients have abnormal neural activity alterations, particularly in brain regions related to learning, memory, and emotion (2). T2DM patients often experience changes in gray matter volume, which can manifest as both functional and structural alterations in the brain (3–8). Our previous study showed brain vascular changes in T2DM patients with carotid artery plaques (9). Diabetes can cause microvascular damage (microangiopathy), affecting small blood vessels in the brain (10, 11). This could lead to changes in the gray-white matter junction due to altered blood flow, ischemia (restricted blood supply), or small vessel disease, potentially resulting in increased tissue volume as a response to these vascular insults. The gray-white matter junction tissue volume (gwJTV) in the brain, which is a novel imaging marker, refers to the region where the gray matter (composed mainly of neuronal cell bodies) transitions into the white matter (composed mainly of myelinated axon tracts). This junction is intricate neural connections and pathways and is crucial for the brain’s overall functionality, as it supports the integration and coordination of neural activities. This gwJTV reduction shows patients with Alzheimer’s disease and correlates with cognitive decline and age (12). Studies have shown that focal changes in the gray-white junction are often present in patients with focal cortical dysplasia (13, 14) and in patients with traumatic brain injury (15). Chronic hyperglycemia can lead to glucose toxicity, which damages neuronal and glial cells (16, 17). Hyperglycemia can also cause osmotic imbalances, leading to cellular swelling and edema. This can contribute to an increase in tissue volume at the gray-white matter junction. Altered insulin signaling in the brain might lead to structural changes, including increased gwJTV, as the brain attempts to adapt to metabolic dysregulation. In our understanding, there are no studies to evaluate gwJTV in the brain of diabetic patients. The gwJTV is being explored as a potential biomarker for early diagnosis and monitoring of neurological diseases.

Although several studies have investigated gray matter volume changes in T2DM patients, there are no studies to investigate the gwJTV damage in diabetes, especially with carotid atherosclerosis (CA). In addition, the mechanisms behind gwJTV damage in diabetes are not understood. Therefore, this study aimed 1) to examine gwJTV difference in the groups of T2DM patients with or without carotid artery plaques and 2) to investigate the associations between the gwJTV and various metabolic parameters related to type 2 diabetes across the whole brain, and then identify the brain structures involved, followed by analysis within pre-determined specific brain areas.

## 2 Methods and materials

### 2.1 Participants

We prospectively recruited 101 T2DM patients in the outpatient clinic of our institutional hospital from June 2020 to December 2021. This study was approved by our Institutional Review Board (IRB KHNMC 2020-025). All participants gave informed consent. Inclusion criteria were as follows: 1) patients diagnosed with type 2 diabetes at least 6 months before the start of the study; 2) age ranged from 30 to 70 years; 3) body mass index (BMI) under 40 kg/m^2^; and 4) well-controlled blood pressure (systolic blood pressure (SBP) under 140 mmHg and diastolic blood pressure (DBP) under 90 mmHg) and low-density lipoprotein (LDL)-cholesterol <100 mg/dL). Exclusion criteria were: 1) history or clinical signs of cerebrovascular disease; 2) a history of addiction to alcohol or drug, 3) estimated glomerular filtration rate (eGFR) < 30 ml/min/1.73 m^2^; 4) glycated hemoglobin (HbA1c) level exceeding 10.0%; and 5) discrete brain parenchymal lesion such as tumor, hemorrhage, or infarction or contusion on brain MRI. Finally, a total of 69 T2DM patients were enrolled in this cross-sectional and case-control study after excluding those who withdrew the study consent (N = 10), those who refused to take an MRI (N = 16), and those who did not complete an MRI scan (N = 6) due to claustrophobia. A total of 69 participants consisting of 33 participants without plaque and 36 participants with plaque were finally included in the study.

We recorded the demographic information of each patient: age, sex, duration of diabetes, smoking status, past medical history, duration of diabetes (DMdur, years), and current use of medications. BMI was calculated as weight divided by the square of height (kg/m^2^), BP was recorded using an automated sociometric sphygmomanometer on the right arm with the subject in the sitting position, and the Korean version of the Mini-Mental Status Examination (MMSE) score was used to assess cognitive function.

In addition, a non-invasive screening device (VP-1000; Colin Medical Technology Corp., Komaki, Japan) was used to measure the ankle-brachial index (ABI). For the right ABI (RtABI), the highest systolic pressure in the right ankle is divided by the highest systolic pressure in either arm (brachial). For the right or left ABI (LtABI), the highest systolic pressure in the right or left ankle (anterior tibial/dorsalis pedis or posterior tibial) is divided by the highest systolic pressure in either arm.

### 2.2 Biomarkers derived from blood and urine

After overnight fasting for 12 hours, blood samples were collected and analyzed. Levels of blood urea nitrogen (BUN) and creatinine (Cr), as well as alanine aminotransferase (ALT) and aspartate aminotransferase (AST), were measured using a Hitachi 747 automated analyzer. The estimated glomerular filtration rate (GFR1) was calculated using the Modification of Diet in Renal Disease (MDRD) equation. High-sensitivity C-reactive protein (hsCRP) concentrations were measured by immunoradiometric assay (ADVIA 1650; Bayer Diagnostics, Tarrytown, NY, USA). The plasma concentrations of fasting blood sugar (fasting plasma glucose) (FBS(FPG)), total cholesterol (TC), triglycerides (TG), high-density lipoprotein cholesterol (HDL-C), and low-density lipoprotein cholesterol (LDL-C) were measured enzymatically using a 747 Chemistry Analyzer (Hitachi, Tokyo, Japan). Glycosylated hemoglobin (HbA1c) levels were measured by high-performance liquid chromatography (VARIANT II; Bio-Rad Laboratories, Hercules, CA, USA). The plasma lipoprotein(a) (Lpa) concentrations were measured with a latex agglutination immunoassay (Daiichi Pure Chemicals Co., Ltd., Tokyo, Japan). apolipoprotein B (ApoB) and apolipoprotein A1 (ApoA1) were measured using the 7080 chemical analyzer (Hitachi), and then calculated as ratio (ApoA1/ApoB ratio)(apolipoprotein A1/B ratio (BA1ratio). Fasting C-peptide (Cpep) and log C-reactive protein (logcrp) levels were measured using a double-antibody radioimmunoassay (DiaSorin, Stillwater, MN, USA). Plasma insulin concentrations were determined using a radioimmunoassay kit (Linco Research, St. Charles, MO, USA). To estimate insulin sensitivity, the homeostasis model assessment of insulin resistance (HOMA-IR) was calculated using the formulae: [HOMA-IR = glucose (mg/dL) × insulin (µU/mL)/405]. Urinary albumin (mg/dL) and creatinine (mg/dL) were measured from a spot urine sample, and then calculated as a ratio (Urine albumin to creatinine ratio (ACR)).

### 2.3 Determination of the presence of CAS

To obtain information on the presence of carotid atherosclerosis (CAS), a carotid duplex ultrasound was performed by a certified sonographer using an ultrasound device equipped with a high-resolution linear-array transducer (LOGIQ 7; GE, Milwaukee, WI, USA). The thickness of the carotid intima-media was measured on the posterior far wall of both carotid arteries. At least four measurements were taken, each about 1 cm proximal to the bifurcation. The mean and maximum thickness values were calculated using the leading edge-to-edge method. Carotid plaque was defined as a focal structure encroaching into the arterial lumen of at least 0.5 mm or 50% of the surrounding thickness value or a demonstrating thickness *>* 1.5 mm as measured from the media-adventitia interface to the intima-lumen interface (9). The presence of CAS was defined by an increase in mean thickness ≥ 1.0 mm or the presence of carotid plaque (9, 18).

### 2.4 MRI acquisition and imaging processing

A sagittal structural three-dimensional (3D) T1-weighted (T1W) image was acquired using a turbo field echo sequence to calculate the gwJTV with the following parameters: repetition time (TR) = 8.1 ms, echo time (TE) = 3.7 ms, flip angle (FA) = 8°, field-of-view (FOV) = 236 × 236 mm^2^, and voxel size =1× 1× 1 mm^3^. In addition, T2-weighted turbo-spin-echo and fluid-attenuated inversion recovery images were acquired for the examination of any malformation of the brain. MRI images were acquired in each subject using a clinical 3 T MRI system (Ingenia, Philips Medical Systems, Best, The Netherlands).

The following image processing was performed using Statistical Parametric Mapping Version 12 (SPM12) software (Wellcome Department of Imaging Neuroscience, University College, London, UK). The 3D T1W image was segmented into gray matter (GM), white matter (WM), and cerebrospinal fluid (CSF), and spatially normalized into the MNI template by the computational anatomy toolbox (CAT12) segmentation tool (19) to obtain brain tissue volumes of gray matter volume (GMV) and white matter volume (WMV) and to calculate gwJTV. The summation of GMV, WMV, and CSF volume calculated the total intracranial volume (TIV).

The following calculation of gwJTV was performed with our in-house code programmed using MATLAB software (MathWorks, Natick, Massachusetts, USA). In addition to obtain gwJTV, information on the segmented GM and WM brain tissue locations was applied to the normalized 3D T1W image to threshold the signal intensity to create a binary map (12, 14). To set lower and upper thresholds of the binary calculation of the gray-white matter junction, a binary map was created with a voxel-based signal intensity (SI) value of normalized 3D T1WI and location information of segmented gray matter and white matter brain tissues based on the following equations:


BTlower=SIGM50Mean+12SIGM502SD

and


BTupper=SIWM50Mean−12SIWM502SD

where BT is the binary threshold for lower and upper limits. $SI_{GM50}^{Mean}$ and $SI_{WM50}^{Mean}$ were average signal intensities over more than 50% volume of gray matter and 50% volume of white matter in the voxel, respectively. $SI_{GM50}^{2SD}$ and $SI_{WM50}^{2SD}$ were dispersion of the signal intensity defined by two standard deviation (SD) in more than 50% gray matter volume and white matter volume, respectively. Lower and upper thresholds were determined by mean and standard deviation (SD) over partial volume percentage of GM and WM volumes. The binary map set thresholds to 1 or 0 for the gray-white matter junction, which is just like to line that some voxels have no values, differs the line shape among the participants, and does not have information of distribution. The binary junction map was blurred by applying a 3D convolution filter with a cubic matrix of 5 x 5 x 5 to minimize the variation among the participants and to denoise the image. A gwJTV for each participant, which is the junction tissue volume at the area of the gray-white matter binary junction, was calculated by converting the binary junction map to the tissue volume. After that, GMV, WMV, and gwJTV for each voxel in each participant were divided by TIV values to control the brain size difference in each participant. Finally, brain tissue volumes of GMV, WMV, and gwJTV maps for each participant were smoothed using the Gaussian kernel of 8× 8× 8 mm^3^ full width at half-maximum (FWHM) for the voxel-based statistical analyses.

### 2.5 Statistical analyses

#### 2.5.1 Demographic characteristics and blood parameters

A two-sample t-test was performed to evaluate the group difference of age, MMSE, and each biomarker between participants with and without plaque. In the following analyses, we used age as a covariate because the rationale for performing correlations with age stems from the well-established influence of aging on both brain structure and metabolic processes. The degree of relationship between age and levels of each metabolic parameter was analyzed using the Pearson correlation analysis. In addition, the heatmap analysis with Pearson correlation was performed to evaluate the correlation between parameters.

#### 2.5.2 Voxel-based analyses in the whole brain

Voxel-based analysis (VBA) is a neuroimaging analysis technique that provides a powerful tool for identifying localized changes in brain structure and function across different groups of subjects or conditions with a given statistical threshold. Two voxel-based analyses were performed using Statistical Parametric Mapping Version 12 (SPM12) software (Wellcome Department of Imaging Neuroscience, University College, London, UK). First, the group difference of GMV, WMV, and gwJTV between participants without and with plaque was evaluated by a voxel-based two-sample t-test with age as a covariate. Second, the relationship between brain tissue volume loss and the level of each parameter in all participants was assessed by performing voxel-based multiple regression analysis for GMV, WMV, and gwJTV, separately. In this analysis, the dependent variable was the voxel value of each brain tissue volume, while the independent variable was the level of each parameter within the framework of a general linear model that was adjusted for the participant’s age. The negative or positive association of each brain tissue volume with the levels of each parameter was evaluated. A significance level of α = 0.0005 was applied without correction for multiple comparisons and a minimum cluster size of at least 100 contiguous voxels. These voxel-based analyses were performed to define some specific brain areas.

#### 2.5.3 Specific brain region-of-interest-based analyses

For ROI-based analysis of the three brain tissue volumes, specific brain areas (e.q. ROIs) were defined as two different methods. First, specific brain areas were defined at the clusters which were shown in significant association between brain tissue volumes and the metabolic parameters on the results of voxel-based multiple regression analyses listed in **Supplementary Table S1**. Second, additional specific brain areas were defined at the hippocampus, thalamus, middle temporal gyrus, and superior frontal gyrus which showed a significant brain tissue volume loss in T2DM patients shown in previous studies (**Supplementary Table S1**) (3–5, 7, 20–22). GMV, WMV, and gwJTV values for each ROI were extracted from all participants using the Marsbar software (Matthew Brett, http://marsbar.sourceforge.net). The following analyses were performed.

First, the two-sample t-test was used to compare GMV, WMV, and gwJTV values between participants without and with a plaque for each ROI. The statistical power for an independent t-test was calculated using the observed mean difference and the group-level standard deviation of the paired differences with R software. Second, the Pearson partial correlation analysis was performed to investigate the relationship between the values of three brain tissue volumes and the levels of metabolic parameters using the participant’s age as the control factor for each ROI. The statistical power for a partial correlation was calculated using the noncentral t-distribution formula with R software. In addition, a multiple-regression analysis was performed to evaluate the associations between one of the brain tissue volumes and parameters for each ROI. The initial model was “ROI tissue volume = β1*age+ β2*HbA1c +•••+ βN*metabolic parameters + error”. Each brain tissue volume in each ROI was described as a dependent variable, and biomarkers were described as independent variables. The variable was entered into the model if the p-value of each dependent variable was less than 0.05, while the variable was removed from the model if the p-value of each dependent variable was greater than 0.1. The multiple regression model included metabolic parameters showing a significant correlation with brain tissue volumes. In the model, we always included both age and HbA1c. Furthermore, this analysis was repeated separately for the participant groups with and without plaques. The initial input data to the model was the same as the one-group analysis in Step 1. If we find any significant association parameters in Step 1, we do an association task again with only the significant parameters. For the MRI measures, a p-value of 0.016 was used to determine statistical significance. This value was obtained by applying the Bonferroni correction, dividing the conventional threshold of p=0.05 by three, corresponding to the three brain tissue volumes of GMV, WMV, and gwJTV. The Medcalc statistical program (MedCalc Software, Acacialaan, Ostend, Belgium) performed all statistical analyses, except for the heatmap analysis for which R software (ver 4.2.3) was used.

## 3 Result

### 3.1 Demographic characteristics and metabolic parameters

**Table 1** summarizes the information of participants, the average value of each metabolic parameter the results of the group comparison of demographic parameters and metabolic biomarkers between with and without carotid plaques, and the results of the Pearson correlation analysis between age and the level of metabolic biomarkers. All demographic parameters and metabolic parameters were not significantly different between the groups, except the apolipoprotein B/A1 ratio. The results of the heatmap correlation analysis are shown in **Supplementary Figure S1**.

**Table 1:** Demographic information and results of correlation analysis between age and metabolic parameters of type 2 diabetes.

Demographic data	Without plaque	With Plaque	P-value	
No. of subject	33	36	69 (total)	
*Age(years)	56.61 ± 8.18	60.00 ± 7.45	p= 0.076	
#Sex (Male/Female)	22/11 (66.7%/33.3%)	24/12 (66.7%/33.33%)	χ^2^ = 0.278, p= 0.598	
*MMSE (score/30)	27.18 ± 2.90	26.67 ± 1.621	p= 0.373	
Indicators of type 2 diabetes				
Parameters	Without plaque	With plaque	*P-value	Correlation with age
DMdur (years)	8.45 ± 5.29	11.06 ± 6.82	p= 0.083	***r= 0.293, p= 0.014***
BMI (kg/m^2^)	25.61 ± 2.73	25.24 ± 2.77	p= 0.588	r= -0.162, p= 0.183
ALT (IU/L)	29.91 ± 19.62	29.36 ± 2.85	p= 0.902	r= -0.143, p= 0.242
AST (IU/L)	24.45 ± 9.88	26.19 ± 9.72	p= 0.464	r= 0.033, p= 0.788
ApoA1 (mmol/L)	131.68 ± 18.09	127.11 ± 18.87	p= 0.309	r= 0.043, p= 0.726
ApoB (nmol/L)	67.16 ± 13.43	72.92 ± 16.12	p= 0.114	r= -0.190, p= 0.117
BA1ratio	0.52 ± 0.12	0.58 ± 0.13	***p= 0.049***	r= -0.188, p= 0.123
BUN (mg/dL)	16.00 ± 3.98	16.94 ± 5.00	p= 0.391	***r= 0.252, p= 0.037***
Cr (mg/dL)	0.75 ± 0.18	0.76 ± 0.20	p= 0.841	r= 0.148, p= 0.224
ACR (mg/mmol)	18.33 ± 24.01	31.01 ± 45.27	p= 0.147	r= -0.005, p= 0.968
GFR1 (mL/min)	103.45 ± 23.94	102.87 ± 30.97	p= 0.932	***r= -0.261, p= 0.030***
HbA1c (mmol/mol)	7.00 ± 0.87	7.02 ± 0.77	p= 0.945	r= -0.156, p= 0.201
FBS(FPG) (mg/dL)	141.52 ± 45.12	141.31 ± 44.71	p= 0.985	r= -0.106, p= 0.385
HOMAIR	4.39 ± 9.74	6.59 ± 19.31	p= 0.548	r= -0.107, p= 0.380
insulinA (mL)	12.72 ± 32.61	15.86 ± 37.11	p= 0.712	r= -0.122, p= 0.316
Cpep (ng/ml)	1.59 ± 0.70	1.72 ± 0.78	p= 0.463	r= -0.116, p= 0.343
HScrp (mg/L)	0.55 ± 0.42	0.56 ± 0.57	p= 0.955	***r= -0.248, p= 0.040***
logcrp	-0.41 ± 0.42	-0.40 ± 0.35	p= 0.969	r= -0.175, p= 0.151
Lpa (nmol/L)	15.70 ± 21.46	18.07 ± 21.16	p= 0.645	r= 0.017, p= 0.892
logLpa	0.90 ± 0.48	0.90 ± 0.63	p= 0.984	r= 0.0002, p= 0.999
LtABI	1.14 ± 0.09	1.12 ± 0.09	p= 0.342	r= 0.076, p= 0.534
RtABI	1.15 ± 0.08	1.14 ± 0.07	p= 0.790	r= 0.011, p= 0.929
SBP (mmHg)	127.48 ± 11.34	130.44 ± 12.23	p= 0.302	r= 0.202, p= 0.097
DBP (mmHg)	75.42 ± 8.31	75.81 ± 8.05	p= 0.847	r= 0.012, p= 0.923
HDL (mg/dL)	49.58 ± 9.67	49.31 ± 10.69	p= 0.913	r= 0.097, p= 0.426
LDL (mg/dL)	61.12 ± 19.83	65.81 ± 21.44	p= 0.351	r= -0.041, p= 0.740
TC (mg/dL)	134.98 ± 20.39	145.22 ± 28.48	p= 0.093	r= -0.098, p= 0.424
TG (mg/dL)	134.67 ± 78.96	141.39 ± 79.54	p= 0.726	***r= -0.350, p= 0.003***

The value presents mean ± standard deviation (SD) (25^th^ percentile – 75^th^ percentile). The Pearson correlation analysis is used to evaluate the degree of relationship between age and levels of each blood biomarker. *Italic* and bold characters in each column indicate a statistical significance.

ACR, Urine albumin to creatinine ratio; ALT, Alanine aminotransferase; ApoA1, apolipoprotein A1; ApoB, apolipoprotein B; AST, aspartate aminotransferase; BA1ratio, apolipoprotein A1/B ratio; BMI, body mass index; BUN, blood urea nitrogen; Cpep, C-peptide; Cr, creatinine; DBP, diastolic blood pressure; DMdur, duration of diabetes (years); FBS(FPG), Fasting Blood Sugar (Fasting Plasma Glucose); GFR1, glomerular filtration rate; HbA1c, Glycated haemoglobin; HDL, high-density lipoprotein; HOMAIR, Homeostasis model assessment of insulin resistance; HScrp, high-sensitivity C-reactive protein; LDL, low-density lipoprotein; logcrp, log C-reactive protein; Lpa. lipoprotein(a); LtABI, left ankle–brachial index; RtABI, right ankle–brachial index; SBP, systolic blood pressure; TC, total cholesterol; TG, triglyceride.

### 3.2 Voxel-based analyses

**Figure 1:**
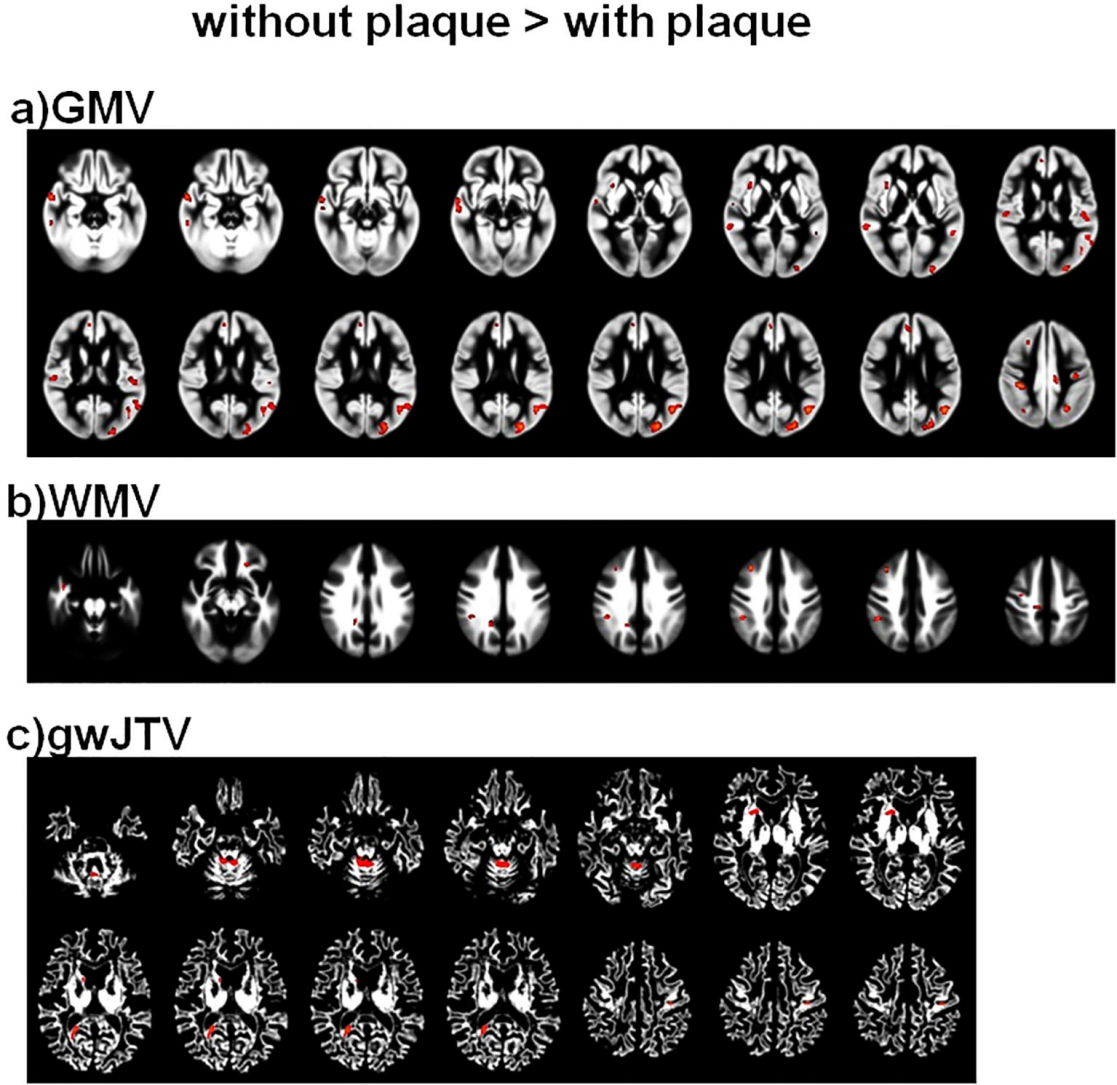
Results of the voxel-based group comparison of GMV **(a)**, WMV **(b)**, and gwJTV **(c)** between participants without and with carotid plaques adjusted for the participant’s age. A significance level of α = 0.0005 was applied without correction for multiple comparisons and a minimum cluster size of at least 100 contiguous voxels. The red indicates a significant group difference between participants without plaque and those with plaque. The detailed association brain areas are listed in **Supplementary Table S2**. GMV, gray matter volume; WMV, white matter volume; gwJTV, gray-white matter junction tissue volume.

**Figure 1** shows the results of the group differences of GMV, WMV, and gwJTV between participants without and with carotid plaques. GMV, WMV, and gwJTV were lower in participants with plaque than in participants without plaque. The red color in **Figure 1** indicates significantly higher brain tissue volumes in participants without plaque than in those with plaque. The detailed brain areas shown in the significant group difference are listed in **Supplementary Table S2** with cluster size, the cluster location, Brodmann area (BA), and Z-score. For gwJTV, the representative brain areas were the postcentral gyrus, caudate body, and cerebellar.

**Figure 2:**
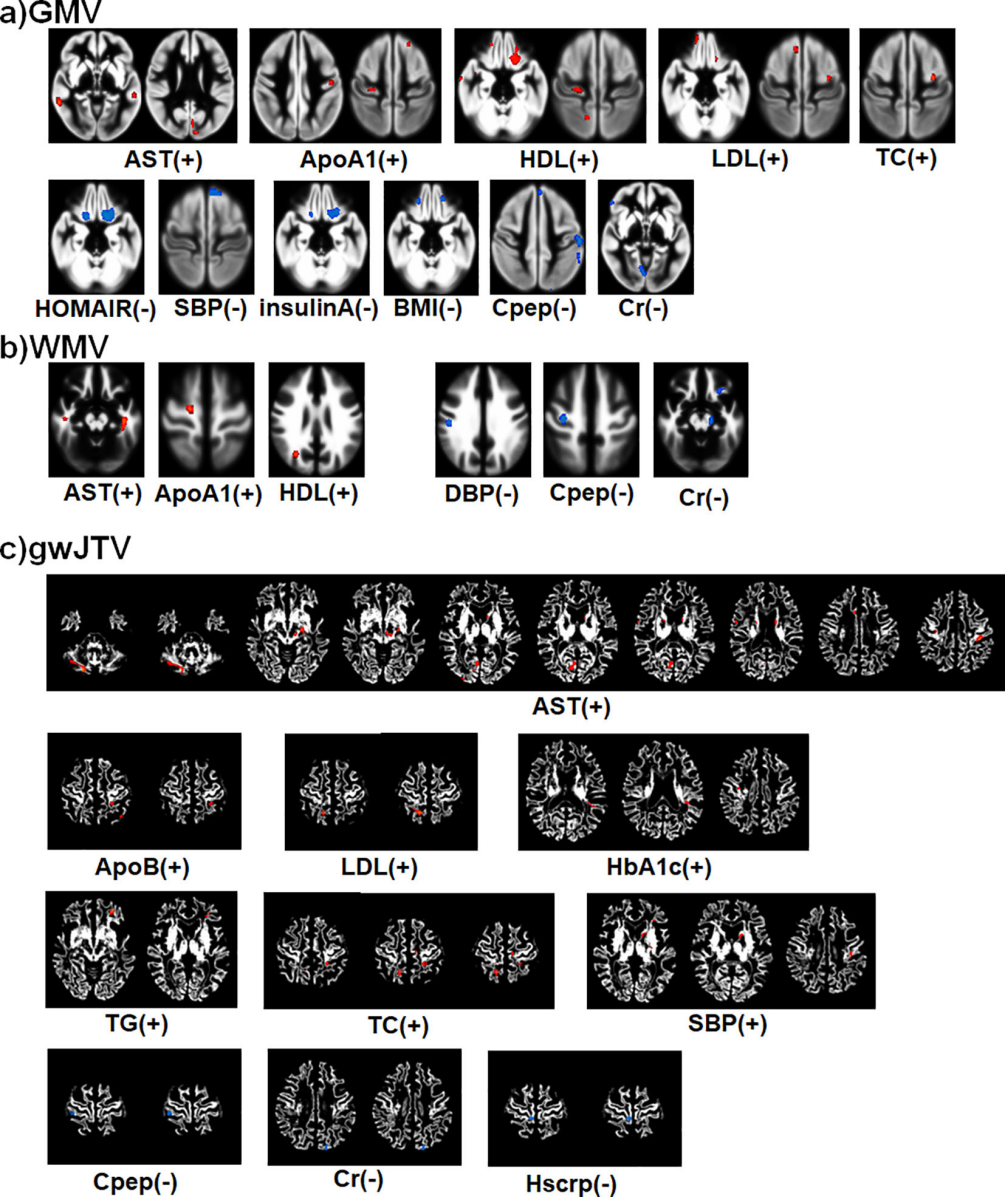
Summary of the voxel-based multiple regression analysis result between brain tissue volumes of GMV **(a)**, WMV **(b)**, and gwJTV **(c)** and metabolic biomarkers adjusted for the participant’s age. A significance level of α = 0.0005 was applied without correction for multiple comparisons and a minimum cluster size of at least 100 contiguous voxels. The colors indicate a positive association with red with the (+) sign and a negative association with blue with the (-) sign. The detailed association brain areas are listed in **Supplementary Table S3** for gwJTV, **Supplementary Table S4** for GMV, and **Supplementary Table S5** for WMV. GMV, gray matter volume; WMV, white matter volume; gwJTV, gray-white matter junction tissue volume; ALT, Alanine aminotransferase; ApoA1, apolipoprotein A1; ApoB, apolipoprotein B; AST, aspartate aminotransferase; BA1ratio, apolipoprotein A1/B ratio; BMI, body mass index; Cr, creatinine; HbA1c, Glycated hemoglobin; HDL, high-density lipoprotein; HOMAIR, Homeostasis model assessment of insulin resistance; HScrp, high-sensitivity C-reactive protein; LDL, low-density lipoprotein; SBP, systolic blood pressure; TC, total cholesterol; TG, triglyceride.

**Figure 2** summarizes the result of the voxel-based multiple regression analysis between brain tissue volumes and metabolic parameters. In the figure, the colors indicate a positive association with red with the (+) sign and a negative association with blue with the (-) sign. First, gwJTV was positively associated with AST, ApoB, LDL, HbA1c, TG, TC, and SBP, but was negatively associated with Cpep, Cr, and Hscrp. The detailed association brain areas are listed in **Supplementary Table S3** with cluster size, the cluster location, Brodmann area (BA), and Z-score. Results are shown as positive (+) and negative (-) association with gwBTV. The representative significant areas were included in the parietal postcentral gyrus, frontal cingulate and precentral gyrus, occipital cuneus, caudate head, temporal gyrus, precuneus, globus pallidus, brainstem midbrain, and cerebellar. Second, GMV was positively associated with the levels of AST, ApoA1, HDL, LDL, and TC, but was negatively associated with the levels of HOMAIR, SBP, Insulin, BMI, Cpep, and Cr. The detailed association brain areas are listed in **Supplementary Table S4**. The representative significant areas were included in the temporal gyrus, occipital cuneus, frontal precentral gyrus, parietal postcentral gyrus, frontal gyrus, precuneus, and cerebellar. Finally, WMV was positively associated with the levels of AST, ApoA1, and HDL, but was negatively associated with the levels of DBP, Cpep, and Cr. The detailed association brain areas are listed in **Supplementary Table S5**. The representative significant areas were included in the temporal WM, parietal WM, frontal WM, cingulate WM, occipital lingual and cuneus WM, precuneus WM, brainstem midbrain, and cerebellar WM.

### 3.3 Specific brain region-of-interest-based analyses

**Table 2** lists the results of the group comparison of GMV, WMV, and gwJTV between participants without and with carotid plaques. The statistical power results for each measurement are also listed in **Table 2**. The values of gwJTV were significantly lower in participants with carotid plaques compared to those without plaque in Cluster 7 (Rt sub-lobar extra-nuclear, p= 0.0004). The values of GMV and WMV were significantly lower in participants with plaque compared to those without carotid plaques in Cluster 6 (Lt middle temporal gyrus, p< 0.0001 for GMV and p= 0.022 for WMV). In addition, we analyzed brain tissue volumes with 54 participants (26 without plaque and 28 with plaque) with excluding 15 participants with MMSE < 26 (7 without plaque and 8 with plaque), the results are nearly identical to those obtained with the original analyses. This result is listed in **Supplementary Table S8**.

**Table 2:** Results of group comparison of three brain tissue volumes between participants with and without plaque in each brain area.

ROIs	Tissue volume	Without plague	With plague	Statistics (p)	Power
Cluster 1 Rt Medial Frontal Gyrus	GMV	0.397 ± 0.066	0.385 ± 0.080	p= 0.498	0.0414
	WMV	0.011 ± 0.008	0.009 ± 0.006	p= 0.224	0.1052
	gwJTV	0.140 ± 0.035	0.135 ± 0.039	p= 0.570	0.0328
Cluster 2 Lt Medial Frontal Gyrus	GMV	0.156 ± 0.055	0.158 ± 0.056	p= 0.900	0.0171
	WMV	0.005 ± 0.004	0.003 ± 0.004	p= 0.175	0.3523
	gwJTV	0.061 ± 0.029	0.061 ± 0.028	p= 0.997	0.0160
Cluster 3 Rt Parietal Lobe	GMV	0.164 ± 0.046	0.164 ± 0.056	p= 0.944	0.0160
	WMV	0.266 ± 0.081	0.250 ± 0.114	p= 0.504	0.0406
	gwJTV	0.184 ± 0.059	0.176 ± 0.056	p= 0.575	0.0341
Cluster 4 Lt Parietal Lobe	GMV	0.190 ± 0.066	0.169 ± 0.055	p= 0.163	0.1588
	WMV	0.236 ± 0.099	0.252 ± 0.097	p= 0.510	0.0415
	gwJTV	0.248 ± 0.057	0.280 ± 0.097	p= 0.088	0.2138
Cluster 5 Left Brainstem Midbrain	GMV	0.027 ± 0.009	0.023 ± 0.010	p= 0.079	0.2402
	WMV	0.380 ± 0.058	0.378 ± 0.052	p= 0.904	0.0172
	gwJTV	0.172 ± 0.063	0.159 ± 0.055	p= 0.354	0.0655
**Cluster 6** Lt Middle Temporal Gyrus	GMV	0.382 ± 0.063	0.317 ± 0.052	***p< 0.0001***	0.9853
	WMV	0.027 ± 0.016	0.041 ± 0.032	***p= 0.022***	1.0000
	gwJTV	0.140 ± 0.031	0.129 ± 0.031	p= 0.166	0.1665
Cluster 7 Rt Sub-lobar Extra-Nuclear	GMV	0.110 ± 0.072	0.111 ± 0.080	p= 0.918	0.0161
	WMV	0.362 ± 0.099	0.352 ± 0.104	p= 0.679	0.0247
	gwJTV	0.417 ± 0.096	0.321 ± 0.117	***p= 0.0004***	0.8881
Hippocampus	GMV	0.364 ± 0.047	0.346 ± 0.060	p= 0.181	0.1446
	WMV	0.060 ± 0.010	0.057 ± 0.012	p= 0.286	0.0952
	gwJTV	0.124 ± 0.023	0.111 ± 0.029	p= 0.056	0.4754
Middle temporal gyrus	GMV	0.293 ± 0.033	0.282 ± 0.031	p= 0.179	0.1558
	WMV	0.090 ± 0.011	0.090 ± 0.015	p= 0.947	1.0000
	gwJTV	0.121 ± 0.014	0.119 ± 0.013	p= 0.574	0.0389
Superior frontal gyrus	GMV	0.197 ± 0.030	0.192 ± 0.027	p= 0.466	0.0459
	WMV	0.054 ± 0.012	0.053 ± 0.011	p= 0.638	0.0228
	gwJTV	0.099 ± 0.018	0.096 ± 0.014	p= 0.622	1.0000
Thalamus	GMV	0.253 ± 0.041	0.247 ± 0.049	p= 0.580	0.0322
	WMV	0.208 ± 0.033	0.198 ± 0.030	p= 0.175	0.1317
	gwJTV	0.455 ± 0.068	0.436 ± 0.068	p= 0.252	0.1015

Data of three brain tissue volumes for each group are presented as mean ± standard deviation. Results of the group comparison of three brain tissue volumes between participants without and with plaque are listed as p-value. *Italic* and bold characters in each column indicate a statistical significance.

*Italic* and bold characters indicate a significant difference of MRI measures between patients with and without plaques. The p-value of 0.016 (p=0.05/3, which are GMV, WMV, and gwJTV) was used to consider statistical significance.

GMV, gray matter volume; WMV, white matter volume; gwJTV, gray-white matter junction tissue volume.

**Table 3** lists the result of the Pearson partial correlation analyses between brain tissue volumes and metabolic parameters in the defined brain area. The statistical power results for each measurement are also listed in **Table 3**. First, gwJTV was positively correlated with ApoA1, HDL, TG, ApoB, LDL, BA1 ratio, and TC, but was negatively correlated with BMI. In detail, gwJTV was positively correlated with ApoB (r= 0.412, p= 0.0005), BA1 ratio (r= 0.329, p= 0.006), LDL (r= 0.346, p= 0.004), and TG (r= 0.460, p= 0.0001) at the left parietal lobe and positively correlated with HDL at the right parietal lobe (r= 0.358, p= 0.003) and with ApoA1 at the superior frontal gyrus (r= 0.316, p= 0.009). Furthermore, gwJTV was negatively correlated with BMI (r= -0.304, p= 0.012). Second, GMV was positively correlated with ApoA1 and HDL but was negatively correlated with HOMAIR, insulin, and BMI. In detail, GMV was positively correlated with HDL at the right medial frontal gyrus (r= 0.299, p= 0.013), left medial frontal gyrus (r= 0.298, p= 0.014), and superior frontal gyrus (r= 0.344, p= 0.004). In addition, GMV was positively correlated with ApoA1 at the superior frontal gyrus (r= 0.352, p= 0.003). Furthermore, GMV was negatively correlated with HOMAIR at the right medial frontal gyrus (r= -0.503, p< 0.0001), insulin at the right medial frontal gyrus (r= -0.425, p= 0.0003), and BMI at the left medial frontal gyrus (r= -0.441, p= 0.0002). Finally, WMV was negatively correlated with Cr at the left brainstem midbrain (r= -0.535, p< 0.0001).

**Table 3:** Results of partial correlation analysis between brain tissue volumes and metabolic parameters in each brain area.

ROIs	biomarkers	GMV(r/p)	WMV(r/p)	gwJTV(r/p)
Cluster 1 Rt Medial Frontal Gyrus	ALT	NS	NS	r= -0.254, p= 0.036 (power=0.374)
	ApoA1	r= 0.251, p= 0.039 (power=0.364)	NS	NS
	HDL	***r= 0.299, p= 0.013*** (power=0.532)	NS	NS
	HOMAIR	***r= -0.503, p< 0.0001*** (power=0.987)	NS	NS
	insulinA	***r= -0.425, p= 0.0003*** (power=0.907)	NS	NS
	BMI	r= -0.269, p= 0.026 (power=0.425)	NS	NS
	DMdur	r= -0.249, p= 0.041 (power=0.357)	NS	NS
Cluster 2 Lt Medial Frontal Gyrus	ALT	NS	r= 0.262, p= 0.031 (power=0.401)	NS
	ApoA1	r= 0.269, p= 0.026 (power=0.425)	r= 0.253, p= 0.038 (power=0.370)	r= 0.266, p= 0.028 (power=0.414)
	HDL	***r= 0.298, p= 0.014*** (power=0.529)	NS	r= 0.272, p= 0.025 (power=0.435)
	BMI	***r= -0.441, p= 0.0002*** (power=0.932)	NS	***r= -0.304, p= 0.012*** (power=0.550)
Cluster 3 Rt Parietal Lobe	ApoA1	NS	NS	r= 0.245, p= 0.044 (power=0.344)
	HDL	NS	NS	***r= 0.358, p= 0.003*** (power=0.738)
	LDL	NS	r= 0.239, p= 0.050 (power=0.325)	NS
	HOMAIR	NS	NS	r= -0.244, p= 0.045 (power=0.341)
	insulinA	NS	NS	r= -0.246, p= 0.043 (power=0.347)
	BMI	NS	NS	***r= -0.418, p= 0.0004*** (power=0.894)
Cluster 4 Lt Parietal Lobe	ApoB	NS	NS	***r= 0.412, p= 0.0005*** (power=0.882)
	BA1ratio	NS	NS	***r= 0.329, p= 0.006*** (power=0.640)
	LDL	NS	NS	***r= 0.346, p= 0.004*** (power=0.699)
	TC	NS	NS	***r= 0.460, p= 0.0001*** (power=0.956)
	SBP	r= 0.246, p= 0.044 (power=0.347)	NS	NS
Cluster 5 Left Brainstem Midbrain	Cr	NS	***r= -0.535, p< 0.0001*** (power=0.996)	NS
	HDL	NS	r= 0.252, p= 0.038 (power=0.367)	NS
	HOMAIR	r= -0.249, p= 0.041 (power=0.357)	NS	NS
	insulinA	r= -0.257, p= 0.035 (power=0.384)	NS	NS
Cluster 6 Lt Middle Temporal Gyrus	logLpa	r= 0.271, p= 0.025 (power=0.432)	NS	NS
Cluster 7 Rt Sub-lobar Extra-Nuclear	GFR1	r= -0.242, p= 0.046 (power=0.355)	NS	NS
Hippocampus	AST	NS	r= 0.289, p= 0.017 (power=0.496)	NS
	HOMAIR	r= -0.254, p= 0.036 (power=0.374)	r= -0.259, p= 0.033 (power=0.390)	r= -0.239, p= 0.050 (power=0.325)
Middle temporal gyrus	AST	r= 0.273, p= 0.024 (power=0.439)	NS	NS
Superior frontal gyrus	ApoA1	***r= 0.352, p= 0.003*** (power=0.719)	NS	***r= 0.316, p= 0.009*** (power=0.594)
	Cpep	r= -0.288, p= 0.017 (power=0.492)	NS	NS
	HDL	***r= 0.344, p= 0.004*** (power=0.692)	NS	r= 0.285, p= 0.019 (power=0.481)
Thalamus	AST	NS	NS	r= 0.259, p= 0.033 (power=0.390)

Results of Partial correlation analysis between brain tissue volume and three brain tissue volumes with covariates as participant’s age are listed as correlation coefficient (r) and p-value. The cluster ROI areas are summarized in **Supplementary Table S1**. *Italic* and bold characters indicate a significant correlation between MRI measures and biomarkers with p=0.016 (p=0.05/3, which are GMV, WMV, and gwJTV). The “not significance (NS)” was indicated with p>0.05.

BTV, brain tissue volume; GMV, gray matter volume; WMV, white matter volume; gwJTV, gray-white matter junction tissue volume; ACR, Urine albumin to creatinine ratio; ALT, Alanine aminotransferase; ApoA1, apolipoprotein A1; ApoB, apolipoprotein B; AST, aspartate aminotransferase; BA1ratio, apolipoprotein A1/B ratio; BMI, body mass index; BUN, blood urea nitrogen; Cpep, C-peptide; Cr, creatinine; DBP, diastolic blood pressure; DMdur, duration of diabetes (years); FBS(FPG), Fasting Blood Sugar (Fasting Plasma Glucose); GFR1, glomerular filtration rate; HbA1c, Glycated hemoglobin; HDL, high-density lipoprotein; HOMAIR, Homeostasis model assessment of insulin resistance; HScrp, high-sensitivity C-reactive protein; LDL, low-density lipoprotein; logcrp, log C-reactive protein; Lpa. lipoprotein(a); LtABI, left ankle–brachial index; RtABI, right ankle–brachial index; SBP, systolic blood pressure; TC, total cholesterol; TG, triglyceride.

**Table 4** lists the results of the multiple regression analyses using the model described in the method part. First, gwJTV was positively associated with ApoA1 at the superior frontal gyrus (β= 0.0003, p= 0.009) and TC at the left parietal lobe (β= 0.001, p= 0.0001). Second, GMV was positively associated with AST at the superior frontal gyrus (β= 0.0006, p= 0.001), but was negatively associated with HOMAIR at the right medial frontal gyrus (β= -0.002, p< 0.0001) and BMI at the left medial frontal gyrus (β= -0.009, p= 0.0002). Finally, WMV was positively associated with ApoA1 at the left medial frontal gyrus (β= 0.00007, p= 0.016), but was negatively associated with Cr at the left brainstem midbrain (β= -0.157, p< 0.0001).

**Table 4:** Results of multiple regression analysis of brain tissue volumes with metabolic parameters in specific brain areas.

ROIs	BTV	Model inputs	Model excluded	Significant result
Cluster 1 Rt Medial Frontal Gyrus	GMV	Age, HbA1c, ApoA1, HDL, HOMAIR, insulinA, BMI, Mdur	Age, HbA1c, ApoA1, HDL, insulinA, DMdur	HOMAIR (β= -0.002, ***p< 0.0001***) BMI (β= -0.007, p= 0.018)
	WMV	Age, HbA1c	Age, HbA1c	No significance
	gwJTV	Age, HbA1c, ALT	Age, HbA1c	ALT (β= -0.0005, p= 0.047)
Cluster 2 Lt Medial Frontal Gyrus	GMV	age, HbA1c, ApoA1, HDL, BMI	age, HbA1c, ApoA1, HDL,	BMI (β= -0.009, ***p= 0.0002***)
	WMV	age, HbA1c, ALT, ApoA1	Age, HbA1c	ALT (β= 0.00007, p= 0.017) ApoA1 (β= 0.00007, ***p= 0.016***)
	gwJTV	Age, HbA1c, ApoA1, HDL, BMI	Age, HbA1c, ApoA1, HDL,	BMI (β= -0.003, p= 0.011)
Cluster 3 Rt Parietal Lobe	GMV	Age, HbA1c	Age, HbA1c	No significance
	WMV	Age, HbA1c, LDL	Age, HbA1c	LDL (β= 0.001, p= 0.046)
	gwJTV	Age, HbA1c, ApoA1, HDL, HOMAIR, insulinA, BMI	Age, HbA1c, ApoA1, HOMAIR, insulinA, BMI	HDL (β= 0.001, p= 0.023)
Cluster 4 Lt Parietal Lobe	GMV	Age, HbA1c, SBP	Age, HbA1c	SBP (β= 0.001, p= 0.035)
	WMV	Age, HbA1c	Age, HbA1c	No significance
	gwJTV	Age, HbA1c, ApoB, BA1ratio, LDL, TC	Age, HbA1c, ApoB, BA1ratio, LDL	TC (β= 0.001, ***p= 0.0001***)
Cluster 5 Left Brainstem Midbrain	GMV	Age, HbA1c, HOMAIR, insulin A	Age, HbA1c, HOMAIR, insulin A	No significance
	WMV	Age, HbA1c, Cr, HDL	Age, HbA1c, HDL	Cr (β= -0.157, ***p< 0.0001***)
	gwJTV	Age, HbA1c	Age, HbA1c	No significance
Cluster 6 Lt Middle Temporal Gyrus	GMV	Age, HbA1c, logLpa	HbA1c, logLpa	Age (β= -0.002, p= 0.021)
	WMV	Age, HbA1c	Age, HbA1c	No significance
	gwJTV	Age, HbA1c	Age, HbA1c	No significance
Cluster 7 Rt Sub-lobar Extra-Nuclear	GMV	Age, HbA1c, GFR1	Age, HbA1c, GFR1	No significance
	WMV	Age, HbA1c	Age, HbA1c	No significance
	gwJTV	Age, HbA1c	Age, HbA1c	No significance
Hippocampus	GMV	Age, HbA1c, HOMAIR	HbA1c	Age (β= -0.0019, p= 0.019) HOMAIR (β= -0.0009, p= 0.036)
	WMV	Age, HbA1c, AST, HOMAIR	HbA1c	Age (β= -0.0005, ***p= 0.001***) AST (β= 0.0003, p= 0.022) HOMAIR (β= -0.0002, p= 0.043)
	gwJTV	Age, HbA1c, HOMAIR	HbA1c	Age (β= -0.0016, ***p= 0.0001***) HOMAIR (β= -0.0004, p= 0.050)
Middle temporal gyrus	GMV	Age, HbA1c, AST	HbA1c	Age (β= -0.001, p= 0.035) AST (β= 0.0009, p= 0.024)
	WMV	Age, HbA1c	Age, HbA1c	No significance
	gwJTV	Age, HbA1c	Age, HbA1c	No significance
Superior frontal gyrus	GMV	Age, HbA1c, ApoA1, HDL	Age, HDL	HbA1c (β= 0.008, p= 0.043) AST (β= 0.0006, ***p= 0.001***)
	WMV	Age, HbA1c	Age, HbA1c	No significance
	gwJTV	Age, HbA1c, ApoA1, HDL	Age, HbA1c, HDL	ApoA1 (β= 0.0003, ***p= 0.009***)
Thalamus	GMV	Age, HbA1c	Age, HbA1c	No significance
	WMV	Age, HbA1c	HbA1c	Age (β= -0.0015, ***p= 0.002***)
	gwJTV	Age, HbA1c, AST	Age, HbA1c	AST (β= 0.0017, p= 0.038)

Results of stepwise multiple regression analysis for the association between brain tissue volumes and blood biomarkers are listed as correlation coefficient (β) and p-value. The parameters input in the model were determined by results of partial correlation analysis. The initial model is ROI tissue volume = β1*age+ β2*HbA1c+…+ βn* metabolic parameter+ error. The variables were included in the model with p< 0.05 and excluded in the model with p> 0.1. The cluster ROI areas are summarized in **Supplementary Table S1**. *Italic* and bold characters indicate a significant correlation between MRI measures and biomarkers with p=0.016 (p=0.05/3, which are GMV, WMV, and gwJTV).

BTV, brain tissue volume GMV, gray matter volume; WMV, white matter volume; gwJTV, gray-white matter junction tissue volume; ALT, Alanine aminotransferase; ApoA1, apolipoprotein A1; ApoB, apolipoprotein B; AST, aspartate aminotransferase; BA1ratio, apolipoprotein A1/B ratio; BMI, body mass index; Cr, creatinine; DMdur, duration of diabetes (years); HbA1c, Glycated hemoglobin; HDL, high-density lipoprotein; HOMAIR, Homeostasis model assessment of insulin resistance; HScrp, high-sensitivity C-reactive protein; LDL, low-density lipoprotein; SBP, systolic blood pressure; TC, total cholesterol; TG, triglyceride.

Results of separate analyses of the participant groups with and without plaques were listed in **Supplementary Table S6** for the participants without plaques and **Supplementary Table S7** for the participants with plaques. Without plaques, gwJTV was significantly positively associated with HDL (β= 0.001, p= 0.009), but negatively associated with BMI (β= -0.011, p= 0.003) and age (β= -0.002, p= 0.0002). GMV was significantly positively associated with ApoA1 (β= 0.001, p= 0.005), but negatively associated with BMI (β= -0.011, p=0.005) and duration (β= -0.005, p= 0.007). WMV was significantly negatively associated with Cr (β= -0.203, p= 0.0001). With plaques, gwJTV was significantly positively associated with TC (β= 0.002, p= 0.001), but negatively associated with age (β= -0.004, p= 0.001). GMV was significantly positively associated with logLpa (β= 0.040, p= 0.003), but negatively associated with HOMAIR (β= -0.002, p= 0.0001). WMV was significantly negatively associated with Cr (β= -0.124, p=0.003).

## 4 Discussion

In this study, we investigated the group difference of gwJTV, which is a new MRI measure to understand the integrity of the junction between gray matter and white matter in the brain of T2DM in patients with or without carotid atherosclerosis and investigated the association between metabolic parameters and brain tissue volume indices, including gwJTV. We found that the gwJTV values were lower in participants with carotid artery plaques compared to those without plaque and found a positive association between brain tissue volumes and ApoA1, AST, HDL, LDL, TC, and SBP and a negative association between brain tissue volumes and HOMAIR, Cr, and BMI. In particular, the voxel-based result showed that gwJTV was positively associated with AST, ApoB, LDL, HbA1c, TG, TC, and SBP, but negatively associated with C-peptide, Cr, and hsCRP. Our findings indicate that maintaining healthy levels of some metabolic parameters such as ApoA1, AST, cholesterol levels, HOMAIR, Cr, and BMI could benefit brain health in T2DM patients.

### 4.1 gwJTV difference in the T2DM patients with and without carotid atherosclerosis

We showed that GMV, WMV, and gwJTV were significantly smaller in the T2DM patients with carotid plaques than those without those. Type 2 diabetes is associated with micro- and macro-vascular complications, which can be exacerbated by the presence of carotid plaques (4, 9). Carotid plaques are a sign of atherosclerosis, which can lead to reduced blood flow to the brain (23). This reduced cerebral blood flow can result in chronic hypoperfusion (insufficient blood supply), causing neuronal and glial cell damage and loss, and can contribute to cell death and atrophy in both gray and white matter regions (24). which manifests as reduced GMV, WMV, and gwJTV. Small vessel disease can cause microinfarcts and diffuse white matter changes, leading to a reduction in WMV and gwJTV. Atherosclerosis and diabetes can compromise the blood-brain barrier, leading to increased permeability, inflammation, and subsequent neuronal and axonal damage (25). Both type 2 diabetes and atherosclerosis are associated with chronic systemic inflammation (17, 26). This inflammation can extend to the brain, contributing to neuroinflammatory processes that lead to tissue damage and volume loss in the gray and white matter, including the gwJTV. Patients with type 2 diabetes and carotid plaques are likely to experience higher levels of oxidative stress (27). Oxidative stress can damage cellular structures in the brain, leading to neuronal and glial cell loss and reduced brain volumes. Type 2 diabetes can cause metabolic dysregulation in the brain, further exacerbating the effects of reduced blood flow and contributing to tissue volume loss (28). Therefore, the presence of carotid plaques in type 2 diabetic patients likely exacerbates vascular, inflammatory, and metabolic challenges, leading to more pronounced brain atrophy and reductions in GMV, WMV, and gwJTV. This highlights the importance of managing both metabolic and vascular health to mitigate the impact on brain structure and function.

Studies have found that T2DM patients can exhibit reduced gray matter volume in various brain regions linked to sensorimotor and visual functions (4), particularly in regions related to cognitive functions and memory (5, 6). Additionally, type 2 diabetes is associated with cortical and subcortical atrophy (7), affecting several brain regions, and diminished regional cerebral perfusion and vasoreactivity, meaning that the blood flow and its responsiveness to changes in carbon dioxide levels are reduced (3). The brain volume changes observed in T2DM patients are linked to cognitive deficits, which may not be fully explained by these structural changes alone. Other factors, such as metabolic disturbances and blood flow dysregulation, particularly affecting the frontal and temporal regions, may also play a role (5). In T2DM patients, an increase in gwJTV in certain brain areas could signify several underlying processes or conditions. Increased gwJTV could reflect neuroinflammatory processes where inflammatory cells and molecules accumulate, potentially leading to localized swelling and increased tissue volume (26). An increase in gwJTV might be a compensatory response to initial cellular damage, where the brain attempts to repair or reorganize affected areas.

We thought that hyperglycemia and its associated metabolic effects could lead to micro- and macro-vascular damage, which in turn affects brain tissue integrity, especially the junction of the gray matter and white matter area where the vascular supply is usually very limited. As age is a known factor in brain atrophy, cognitive decline, and changes in metabolic markers such as insulin resistance, cholesterol levels, and kidney function, it is crucial to control for or examine its impact in studies involving brain integrity and diabetes. In this study, we accounted for age-related changes, ensuring that any observed associations between gwJTV and metabolic parameters are not confounded by age effects.

### 4.2 Positive association between gwJTV and some metabolic parameters

In T2DM patients, the result of ROI-based multiple regression analysis (**Table 4**) showed that gwJTV was positively associated with ApoA1 and TC. GMV was positively associated with AST. WMV was positively associated with ApoA1. ApoA1 is positively associated with brain tissue volumes due to its beneficial roles in lipid metabolism and neuroprotection. ApoA1, a component of HDL, is crucial for lipid metabolism and neuroprotection. It aids in reverse cholesterol transport, potentially maintaining brain structure and protecting against cognitive decline related to diabetes (29). Higher ApoA1 levels correlate with greater brain tissue volumes, which is significant in neurodegenerative conditions like Alzheimer’s disease. (29). ApoA1 may help maintain brain structure integrity by modulating lipid metabolism and protecting neuronal cells from oxidative stress (30, 31).

AST, an enzyme involved in amino acid metabolism, is found in various tissues, including the liver and brain. There is evidence suggesting that elevated AST levels can be indicative of neuronal damage or inflammation, therefore, we expect to potentially lead to reduced brain tissue volumes. A previous study found that conditions like nonalcoholic fatty liver disease (NAFLD) have been linked to cognitive decline and could be reflected in AST levels (32). We can expect that metabolic disturbances might lead to brain atrophy in Type 2 diabetes, (3). Therefore, the specific association between AST levels and brain tissue volumes in T2DM patients is not well established and may require further research for a definitive understanding.

Dyslipidemia plays a very important role in the development of atherosclerosis in patients with T2DM. In T2DM patients, lipid profiles such as HDL, LDL, and TC provide insights into brain health. The result of ROI-based multiple regression analysis (**Table 4**) showed that gwJTV shows a positive association with TC (β= 0.001, p= 0.0001) in the left parietal lobe. In addition, the correlation analysis (**Table 3**) showed that gwJTV was positively correlated with LDL (r= 0.346, p= 0.004) in the left parietal lobe, HDL (r= 0.358, p= 0.003) in the right parietal lobe, and TC (r= 0.460, p= 0.0001) in the left parietal lobe. The result of the voxel-based multiple regression analysis (**Figure 2**) showed that gwJTV shows a positive association with LDL in the right parietal precuneus and TC in the right parietal precuneus and the left frontal paracentral gyrus. We expect a negative correlation between brain tissue volumes and cholesterol levels and summarize our results with some supporting references. HDL has a positive association with brain tissue volumes due to its protective roles, while the association of LDL and TC with brain health is more complex and requires further investigation. We found that higher HDL levels are linked to larger brain tissue volumes. Previous studies were found that higher levels of HDL have been associated with a lower risk of cognitive decline, which suggests a positive association with brain tissue volumes (33, 34). In Type 2 diabetes, the relationship between LDL and brain tissue volumes is complex. Adequate levels of cholesterol are necessary for the brain, and TC levels within a healthy range may be associated with better brain tissue volumes and cognitive function (35), but this area requires further research for a clear understanding.

### 4.3 Negative association between gwJTV and some metabolic parameters

In T2DM patients, the result of ROI-based multiple regression analysis (**Table 4**) showed that GMV was negatively associated with HOMA-IR and BMI. WMV was negatively associated with Cr. The result of the voxel-based multiple regression analysis (**Figure 2**) showed that gwJTV had a significantly negative association with Cpep, Cr, and HS-CRP. We thought the negative association between gwJTV and Cr was reasonable. Creatinine, a waste product indicating kidney function, when elevated, suggests kidney dysfunction. It can lead to reduced gwJTV in the left occipital cuneus and reduced WMV in the left brainstem midbrain (β= -0.157, p< 0.0001). A previous study also showed cognitive impairment and reduced brain tissue volumes (36), accumulation of toxins damaging brain cells (3), and neurological complications from acute kidney injury (AKI) (37) with increased Cr. The metabolic and inflammatory changes from kidney dysfunction contribute to prolonged brain injury (37).

HOMA-IR is a measure used to estimate insulin resistance, which is a condition where cells in the body become less responsive to insulin. HOMA-IR is associated with decreased brain tissue volumes of GMV. A previous study also showed decreased brain tissue volumes in the frontal lobe (3, 21, 38). In addition, we also found a marginal negative association between HOMA-IR and GMV (β= -0.0009, p= 0.036), WMV (β= -0.0002, p= 0.043), and gwJTV (β= -0.0004, p= 0.050) in the hippocampus, which was also associated with the increased risk of cognitive decline and dementia (3). In addition, HOMA-IR is associated with impaired cerebral blood flow and vasoreactivity, potentially resulting in atrophy (3, 39).

### 4.4 Current state of research on dyslipidemia and brain tissue damage

Our results revealed unexpected positive associations between gwJTV and lipid levels, specifically LDL and TC. Dyslipidemia is traditionally seen as a risk factor for brain damage and dysfunction. Elevated cholesterol levels help preserve brain tissue volumes amidst the metabolic challenges posed by T2DM to play a role in maintaining or even enhancing brain tissue volumes, especially in specific sub-populations of T2DM patients. In a comprehensive population-based study with extended follow-up, higher LDL levels were linked to larger total brain volumes, particularly in individuals over 53 years, similar to our participants. Conversely, TC levels were not associated with total brain volume but showed a positive association with AD-related regions, varying by different brain areas linked to AD pathology (40). Another study in a non-demented Korean population, higher LDL-c was correlated with greater Aβ uptake and reduced hippocampal volume, which are risk factors in AD (41). An additional study showed that the combination of low LDL-C and hypertension had a more pronounced impact on brain structure, leading to further reductions in grey matter volume and increased white matter lesions (42), indicating that the relationship between brain tissue volume and LDL varies with the involvement of vascular risk factors such as hypertension. Furthermore, a meta-analysis of cohort and case-control studies found that elevated TC levels are significantly associated with a higher risk of cognitive impairment, reinforcing TC’s role in neurodegeneration (43). A study in non-demented elderly Chinese individuals showed that high TC was linked to altered functional connectivity, indicating potential early neural damage (44), suggesting that TC is more related to brain function damage rather than structural damage. Cholesterol is a critical component of cell membranes and is necessary for proper brain function. Adequate cholesterol levels, particularly within a healthy range, may support brain tissue integrity and function. HDL is known for its protective roles in brain health, with higher HDL levels associated with larger brain tissue volumes. The literature on LDL and TC is less clear, with some evidence suggesting potential beneficial roles under certain conditions, such as maintaining cellular structures and supporting cognitive functions. The mechanisms behind gwJTV damage in T2DM with carotid atherosclerosis are not fully understood, and our study did not explore these mechanisms in detail. Therefore, further research is needed to better understand these relationships. Future studies should aim to explore the mechanisms behind these associations and consider other potential confounding factors such as diet, lifestyle, and genetic predispositions.

### 4.5 Limitations of this study

This study has several limitations. *First*, the small sample size of 69 T2DM patients may limit generalizability, and larger cohorts are needed for validation. *Second*, the cross-sectional design restricts causal inferences between carotid plaques and gwJTV changes. Conducted at a single clinic, the study’s findings may not apply broadly due to demographic and clinical variability. *Third*, although we adjusted for age and HbA1c, other factors like sex, BMI, diabetes duration, kidney function, and medication use could act as confounders. Future studies should consider a wider range of covariates to address potential confounding. *Fourth*, we did not validate our gwJTV methodology against other techniques or histopathological data, which could enhance accuracy and reliability. One technique, developed using signal intensity and a brain tissue segmentation method (12, 13) depends on the results of brain tissue segmentation and the threshold of signal intensity. Another technique, called local directional probability optimization (LDPO), was developed to measure the width of the boundary of the gray-white matter within lesional areas (45). This technique depends on the segmentation of the gray-white boundary region at specific lesional areas. A third method, a tessellation approach based on cortical depth, was developed using FreeSurfer (https://surfer.nmr.mgh.harvard.edu/) (46, 47). None of these methods has been validated with histopathological data. However, a previous immunohistochemistry study showed damage to the gray-white matter junction in traumatic brain injury (48). Lastly, a new imaging sequence, which does not rely on brain tissue segmentation, called Edge-Enhancing Gradient Echo (EDGE), was introduced to directly image the gray-white junction (49, 50). In future studies, it would be worthwhile to compare the EDGE method with gwJTV in the same population. *Finally*, we did not evaluate participants for AD or neurodegenerative conditions using specific biomarkers or comprehensive neuropsychological tests. This omission limits the interpretation of gwJTV as a diabetes-specific biomarker, as reductions might reflect AD pathology. However, it’s important to note that we only included three participants in their 70s. We analyzed brain tissue volumes in 54 participants (26 without plaque and 28 with plaque) while excluding 15 participants with an MMSE score below 26 (7 without plaque and 8 with plaque). The results were nearly identical to those obtained in the original analyses (refer to **Supplementary Table S8**), suggesting that gwJTV reductions in this study could indeed reflect diabetic or carotid atherosclerosis pathology in the brain. Future research should integrate AD screenings to exclude neurodegenerative confounders and establish clearer links between gwJTV loss and AD pathology.

## 5 Conclusion

In this study, we investigated the impact of T2DM patients with carotid atherosclerosis on brain tissue volumes, specifically focusing on gwJTV. T2DM participants without carotid plaques exhibited higher gwTJV compared to those with plaques. VBA showed that gwJTV was positively associated with AST, ApoB, LDL, HbA1c, TG, TC, and SBP, but was negatively associated with Cpep, Cr, and Hscrp. ROI analysis showed that gwJTV was positively correlated with ApoA1, HDL, TG, ApoB, LDL, BA1 ratio, and TC, but was negatively correlated with BMI. gwJTV was positively associated with ApoA1. Without plaques, gwJTV was significantly positively associated with HDL, but negatively associated with BMI and age. With plaques, gwJTV was significantly positively associated with TC, but negatively associated with age. Our research suggests that gwJTV could serve as a potential imaging biomarker for monitoring brain health and vascular health in T2DM patients, particularly those with carotid atherosclerosis. Furthermore, this study’s findings suggest that maintaining healthy levels of certain metabolic parameters could be beneficial for brain health in T2DM patients. Further studies are recommended with a large sample size to validate this study.

## Data Availability

The raw data supporting the conclusions of this article will be made available by the authors, without undue reservation.

## References

[1] Yu MG Gordin D Fu J Park K Li Q King GL. Protective factors and the pathogenesis of complications in diabetes. Endocr Rev. (2024) 45:227–52. doi: 10.1210/endrev/bnad030, PMID: 37638875 PMC10911956

[2] Li ZY Ma T Yu Y Hu B Han Y Xie H. Changes of brain function in patients with type 2 diabetes mellitus measured by different analysis methods: A new coordinate-based meta-analysis of neuroimaging. Front Neurol. (2022) 13. doi: 10.3389/fneur.2022.923310, PMID: 36090859 PMC9449648

[3] Last D Alsop DC Abduljalil AM Marquis RP De Bazelaire C Hu K. Global and regional effects of type 2 diabetes on brain tissue volumes and cerebral vasoreactivity. Diabetes Care. (2007) 30:1193–9. doi: 10.2337/dc06-2052, PMID: 17290035 PMC2031924

[4] Burgess J De Bezenac C Keller SS Frank B Petropoulos IN Garcia-Finana M. Brain alterations in regions associated with end-organ diabetic microvascular disease in diabetes mellitus: A UK Biobank study. Diabetes Metab Res Rev. (2024) 40:1–16. doi: 10.1002/dmrr.3772, PMID: 38363054

[5] Espeland MA Bryan RN Goveas JS Robinson JG Siddiqui MS Liu SM. Influence of type 2 diabetes on brain volumes and changes in brain volumes results from the women’s health initiative magnetic resonance imaging studies. Diabetes Care. (2013) 36:90–7. doi: 10.2337/dc12-0555, PMID: 22933440 PMC3526228

[6] Reynolds EL Votruba K Jack CR Beare R Reid RI Preboske GM. Association between brain health outcomes and metabolic risk factors in persons with diabetes. Ann Clin Trans Neurol. (2023) 10:1891–8. doi: 10.1002/acn3.51859, PMID: 37518982 PMC10578900

[7] Zhang TQ Shaw M Cherbuin N. Association between type 2 diabetes mellitus and brain atrophy: A meta-analysis. Diabetes Metab J. (2022) 46:781–802. doi: 10.4093/dmj.2021.0189, PMID: 35255549 PMC9532183

[8] Roy B Choi SE Freeby MJ Kumar R. Microstructural brain tissue changes contribute to cognitive and mood deficits in adults with type 2 diabetes mellitus. Sci Rep. (2023) 13:9636. doi: 10.1038/s41598-023-35522-9, PMID: 37316507 PMC10267112

[9] Jun JE Hwang YC Ahn KJ Chung HY Jahng GH Park S. Association between carotid atherosclerosis and presence of intracranial atherosclerosis using three-dimensional high-resolution vessel wall magnetic resonance imaging in asymptomatic patients with type 2 diabetes. Diabetes Res Clin Pract. (2022) 191:1–6. doi: 10.1016/j.diabres.2022.110067, PMID: 36067918

[10] Stehouwer CDA. Microvascular dysfunction and hyperglycemia: A vicious cycle with widespread consequences. Diabetes. (2018) 67:1729–41. doi: 10.2337/dbi17-0044, PMID: 30135134

[11] Barrett EJ Liu Z Khamaisi M King GL Klein R Klein BEK. Diabetic microvascular disease: an endocrine society scientific statement. J Clin Endocrinol Metab. (2017) 102:4343–410. doi: 10.1210/jc.2017-01922, PMID: 29126250 PMC5718697

[12] Tian Y Oh JH Rhee HY Park S Ryu CW Cho AR. Gray-white matter boundary Z-score and volume as imaging biomarkers of Alzheimer’s disease. Front Aging Neurosci. (2023) 15:1291376. doi: 10.3389/fnagi.2023.1291376, PMID: 38161586 PMC10755914

[13] Huppertz HJ Grimm C Fauser S Kassubek J Mader I Hochmuth A. Enhanced visualization of blurred gray-white matter junctions in focal cortical dysplasia by voxel-based 3D MRI analysis. Epilepsy Res. (2005) 67:35–50. doi: 10.1016/j.eplepsyres.2005.07.009, PMID: 16171974

[14] Huppertz HJ Wellmer J Staack AM Altenmuller DM Urbach H Kroll J. Voxel-based 3D MRI analysis helps to detect subtle forms of subcortical band heterotopia. Epilepsia. (2008) 49:772–85. doi: 10.1111/j.1528-1167.2007.01436.x, PMID: 18047585

[15] Gasparetto EL Rueda Lopes FC Domingues RC Domingues RC. Diffusion imaging in traumatic brain injury. Neuroimaging Clin N Am. (2011) 21:115–25, viii. doi: 10.1016/j.nic.2011.02.003, PMID: 21477754

[16] Lloriãn-Salvador M Cabeza-Fernãndez S Gomez-Sanchez JA de la Fuente AG. Glial cell alterations in diabetes-induced neurodegeneration. Cell Mol Life Sci. (2024) 81:1–18. doi: 10.1007/s00018-023-05024-y, PMID: 38236305 PMC10796438

[17] Vargas-Soria M Garcïa-Alloza M Corraliza-Gõmez M. Effects of diabetes on microglial physiology: a systematic review of *in vitro*, preclinical and clinical studies. J Neuroinflamm. (2023) 20:1–30. doi: 10.1186/s12974-023-02740-x, PMID: 36869375 PMC9983227

[18] Jun JE Kang H Hwang YC Ahn KJ Chung HY Jeong IK. The association between lipoprotein (a) and carotid atherosclerosis in patients with type 2 diabetes without pre-existing cardiovascular disease: A cross-sectional study. Diabetes Res Clin Pract. (2021) 171:1–10. doi: 10.1016/j.diabres.2020.108622, PMID: 33316308

[19] Seiger R Ganger S Kranz GS Hahn A Lanzenberger R. Cortical thickness estimations of FreeSurfer and the CAT12 toolbox in patients with Alzheimer’s disease and healthy controls. J Neuroimaging. (2018) 28:515–23. doi: 10.1111/jon.12521, PMID: 29766613 PMC6174993

[20] Ma T Li ZY Yu Y Hu B Han Y Ni MH. Gray and white matter abnormality in patients with T2DM-related cognitive dysfunction: a systemic review and meta-analysis. Nutr Diabetes. (2022) 12:39. doi: 10.1038/s41387-022-00214-2, PMID: 35970833 PMC9378704

[21] Raiko JRH Tuulari JJ Saari T Parkkola R Savisto N Nuutila P. Associations between brain gray matter volumes and adipose tissue metabolism in healthy adults. Obesity. (2021) 29:543–9. doi: 10.1002/oby.23094, PMID: 33528921

[22] Roy B Ehlert L Mullur R Freeby MJ Woo MA Kumar R. Regional brain gray matter changes in patients with type 2 diabetes mellitus. Sci Rep. (2020) 10:1–13. doi: 10.1038/s41598-020-67022-5, PMID: 32555374 PMC7303156

[23] Cicha I Wørner A Urschel K Beronov K Goppelt-Struebe M Verhoeven E. Carotid plaque vulnerability A positive feedback between hemodynamic and biochemical mechanisms. Stroke. (2011) 42:3502–U268. doi: 10.1161/STROKEAHA.111.627265, PMID: 21998063

[24] Rajeev V Chai YL Poh L Selvaraji S Fann DY Jo DG. Chronic cerebral hypoperfusion: a critical feature in unravelling the etiology of vascular cognitive impairment. Acta Neuropathol Commun. (2023) 11:1–23. doi: 10.1186/s40478-023-01590-1, PMID: 37309012 PMC10259064

[25] Rhea EM Banks WA. Role of the blood-brain barrier in central nervous system insulin resistance. Front Neurosci. (2019) 13. doi: 10.3389/fnins.2019.00521, PMID: 31213970 PMC6558081

[26] Shukla V Shakya AK Perez-Pinzon MA Dave KR. Cerebral ischemic damage in diabetes: an inflammatory perspective. J Neuroinflamm. (2017) 14:21. doi: 10.1186/s12974-016-0774-5, PMID: 28115020 PMC5260103

[27] Mënëgaut L Laubriet A Crespy V Leleu D Pilot T van Dongen K. Inflammation and oxidative stress markers in type 2 diabetes patients with Advanced Carotid atherosclerosis. Cardiovasc Diabetol. (2023) 22:1–10. doi: 10.1186/s12933-023-01979-1, PMID: 37710315 PMC10503074

[28] Zhang S Zhang YY Wen ZG Yang YN Bu TJ Bu XW. Cognitive dysfunction in diabetes: abnormal glucose metabolic regulation in the brain. Front Endocrinol. (2023) 14. doi: 10.3389/fendo.2023.1192602, PMID: 37396164 PMC10312370

[29] Cochran BJ Ong KL Manandhar B Rye KA. APOA1: a protein with multiple therapeutic functions. Curr Atheroscl Rep. (2021) 23:1–10. doi: 10.1007/s11883-021-00906-7, PMID: 33591433

[30] Lee CH Murrell CE Chu A Pan XY. Circadian regulation of apolipoproteins in the brain: implications in lipid metabolism and disease. Int J Mol Sci. (2023) 24:1–22. doi: 10.3390/ijms242417415, PMID: 38139244 PMC10743770

[31] Song F Poljak A Crawford J Kochan NA Wen W Cameron B. Plasma apolipoprotein levels are associated with cognitive status and decline in a community cohort of older individuals. PloS One. (2012) 7:1–11. doi: 10.1371/journal.pone.0034078, PMID: 22701550 PMC3372509

[32] Yokokawa T Sasaki S Sase K Yoshii N Yasuda J Hayashi T. Association of serum brain-derived neurotrophic factor with hepatic enzymes, AST/ALT ratio, and FIB-4 index in middle-aged and older women. PloS One. (2022) 17:1–9. doi: 10.1371/journal.pone.0273056, PMID: 35998179 PMC9398011

[33] Van Valkenburgh J Meuret C Martinez AE Kodancha V Solomon V Chen K. Understanding the exchange of systemic HDL particles into the brain and vascular cells has diagnostic and therapeutic implications for neurodegenerative diseases. Front Physiol. (2021) 12. doi: 10.3389/fphys.2021.700847, PMID: 34552500 PMC8450374

[34] Vitali C Wellington CL Calabresi L. HDL and cholesterol handling in the brain. Cardiovasc Res. (2014) 103:405–13. doi: 10.1093/cvr/cvu148, PMID: 24907980

[35] Biessels GJ Reijmer YD. Brain changes underlying cognitive dysfunction in diabetes: what can we learn from MRI? Diabetes. (2014) 63:2244–52. doi: 10.2337/db14-0348, PMID: 24931032

[36] Song DK Hong YS Sung YA Lee H. Association of serum creatinine levels and risk of type 2 diabetes mellitus in Korea: a case control study. BMC Endocr Disord. (2022) 22:1–7. doi: 10.1186/s12902-021-00915-2, PMID: 34983489 PMC8725385

[37] Husain-Syed F Takeuchi T Neyra JA Ramirez-Guerrero G Rosner MH Ronco C. Acute kidney injury in neurocritical care. Crit Care. (2023) 27:341. doi: 10.1186/s13054-023-04632-1, PMID: 37661277 PMC10475203

[38] Fujita S Mori S Onda K Hanaoka S Nomura Y Nakao T. Characterization of brain volume changes in aging individuals with normal cognition using serial magnetic resonance imaging. JAMA Netw Open. (2023) 6:1–13. doi: 10.1001/jamanetworkopen.2023.18153, PMID: 37378985 PMC10308250

[39] Nguyen TT Hulme J Vo TK Vo GV. The potential crosstalk between the brain and visceral adipose tissue in Alzheimer’s development. Neurochem Res. (2022) 47:1503–12. doi: 10.1007/s11064-022-03569-1, PMID: 35298764

[40] Moazzami K Power MC Gottesman R Mosley T Lutsey PL Jack C, JR.. Association of mid-life serum lipid levels with late-life brain volumes: The atherosclerosis risk in communities neurocognitive study (ARICNCS). Neuroimage. (2020) 223:117324. doi: 10.1016/j.neuroimage.2020.117324, PMID: 32882383 PMC9006082

[41] Kang SH Yoo H Cheon BK Park YH Kim SJ Ham H. Distinct effects of cholesterol profile components on amyloid and vascular burdens. Alzheimers Res Ther. (2023) 15:197. doi: 10.1186/s13195-023-01342-2, PMID: 37950256 PMC10636929

[42] Chung CP Chou KH Peng LN Liu LK Lee WJ Chen LK. Associations between low circulatory low-density lipoprotein cholesterol level and brain health in non-stroke non-demented subjects. Neuroimage. (2018) 181:627–34. doi: 10.1016/j.neuroimage.2018.07.049, PMID: 30053515

[43] Zhao Y Zhang H Cheng J Zou Y Zhang D Duan X. Association between dyslipidaemia and cognitive impairment: A meta-analysis of cohort and case-control studies. J Integr Neurosci. (2024) 23:40. doi: 10.31083/j.jin2302040, PMID: 38419448

[44] Zhang T Li H Zhang J Li X Qi D Wang N. Impacts of high serum total cholesterol level on brain functional connectivity in non-demented elderly. J Alzheimers Dis. (2016) 50:455–63. doi: 10.3233/JAD-150810, PMID: 26682694

[45] Qu X Yang J Ai D Song H Zhang L Wang Y. Local directional probability optimization for quantification of blurred gray/white matter junction in magnetic resonance image. Front Comput Neurosci. (2017) 11:83. doi: 10.3389/fncom.2017.00083, PMID: 28955216 PMC5600984

[46] Uribe C Segura B Baggio HC Abos A Garcia-Diaz AI Campabadal A. Gray/white matter contrast in Parkinson’s disease. Front Aging Neurosci. (2018) 10:89. doi: 10.3389/fnagi.2018.00089, PMID: 29636679 PMC5881246

[47] Salat DH Chen JJ van der Kouwe AJ Greve DN Fischl B Rosas HD. Hippocampal degeneration is associated with temporal and limbic gray matter/white matter tissue contrast in Alzheimer’s disease. Neuroimage. (2011) 54:1795–802. doi: 10.1016/j.neuroimage.2010.10.034, PMID: 20965261 PMC3021138

[48] Alisafaei F Gong Z Johnson VE Dolle JP Smith DH Shenoy VB. Mechanisms of local stress amplification in axons near the gray-white matter interface. Biophys J. (2020) 119:1290–300. doi: 10.1016/j.bpj.2020.08.024, PMID: 33027609 PMC7567985

[49] Middlebrooks EH Lin C Westerhold E Okromelidze L Vibhute P Grewal SS. Improved detection of focal cortical dysplasia using a novel 3D imaging sequence: Edge-Enhancing Gradient Echo (3D-EDGE) MRI. NeuroImage Clin. (2020) 28:102449. doi: 10.1016/j.nicl.2020.102449, PMID: 33032066 PMC7552096

[50] Middlebrooks EH Greco E Zhou X Gupta V Freund BE Agarwal AK. Edge-Enhancing Gradient Echo MRI at 7T for detection of focal cortical dysplasia in epilepsy. NeuroImage Rep. (2023) 3:100187. doi: 10.1016/j.ynirp.2023.100187, PMID: 40567820 PMC12172902

