# Supplementary material for: Association of carotid atherosclerosis with brain tissue integrity and metabolic parameters in type 2 diabetes patients
Source: Front Endocrinol (Lausanne). 2025 Aug 1;16:1586085. doi: 10.3389/fendo.2025.1586085 (PMC12353737; doi:10.3389/fendo.2025.1586085)
Supplement: Supplementary file 1 [file DataSheet1.docx]

**Impact of Carotid Atherosclerosis on Brain Tissue Integrity in Patients with Type 2 Diabetes**

**Supplementary Materials**

- 1. **Supplementary Tables**

**Supplementary Table S1. Information on the specific region-of-interest (ROI) clusters in the brain obtained from the voxel-based analysis**

| **Index** | **voxel-based analysis** | **Cluster Size** | **Z-score** | **Areas in the brain** | **Picture** |
| --- | --- | --- | --- | --- | --- |
| **Correlation Test** | | | | |  |
| **Cluster 1** | HOMAIR with GMV | 546 | 4.24 | Rt Medial Frontal Gyrus | 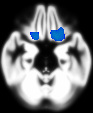 |
| **Cluster 2** | BMI with GMV | 548 | 4.02 | Lt Medial Frontal Gyrus | 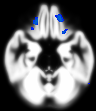 |
| **Cluster 3** | LDL with gwBTV | 508 | 4.97 | Rt Parietal Lobe Precuneus | 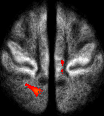 |
|  |  |  | 3.8 | Rt Parietal Lobe Sub-Gyral |  |
| **Cluster 4** | TC with gwBTV | 307 | 3.92 | Lt Parietal Lobe Sub-Gyral | 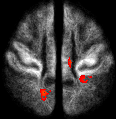 |
| **Cluster 5** | Cr with WMV | 989 | 5.04 | Left Brainstem Midbrain | 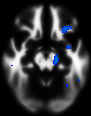 |
| **Group Comparison (without plaque > with plaque)** | | | | |  |
| **Cluster 6** | GMV | 2440 |  | Lt Middle Temporal Gyrus | 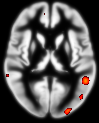 |
| **Cluster 7** | gwJTV | 498 |  | Rt Sub-lobar Extra-Nuclear | 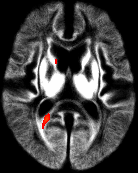 |
| **Specific Brain Region** | | | | | |
| **Hippocampus Middle temporal gyrus Superior frontal gyrus Thalamus**  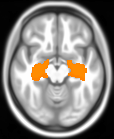 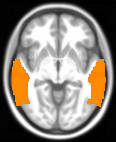 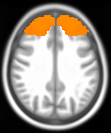 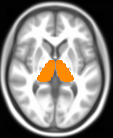 | | | | | |

*Abbreviation*: GMV, gray matter volume; WMV, white matter volume; gwJTV, gray-white matter junction tissue volume; HOMAIR, Homeostasis model assessment of insulin resistance; BMI, body mass index; LDL, low-density lipoprotein; TC, total cholesterol; Cr, creatinine; AST, aspartate aminotransferase; Cpep, C-peptide; DMdur, duration of diabetes (years); HbA1c, Glycated haemoglobin; HDL, high-density lipoprotein; SBP, systolic blood pressure.

**Supplementary Table S2. Significantly different areas of results of voxel-based group comparison of three brain tissue volumes between participants with and without carotid plaques.**

| **Group analysis** | **Cluster size** | **Cluster location** | **BA** | **Talairach coordinates** | **Z score** | | **ROI** |
| --- | --- | --- | --- | --- | --- | --- | --- |
| **Without plaque > with plaque** | | | | | | | |
| **GMV** | 2440 | Lt Middle Temporal Gyrus GM | 21 | -50.29, -46.08, 7.17 | | 5.25 | ROI |
|  | 1926 | Lt Middle Occipital Gyrus GM | 19 | -25.53, -82.66, 14.93 | | 5.07 |  |
|  |  | Lt Occipital Precuneus GM | 31 | -26.52, -73.95, 22.05 | | 3.84 |  |
|  | 542 | Rt Inferior Parietal Lobule GM | 40 | 31.71, -34.56, 40.28 | | 4.97 |  |
|  | 522 | Lt Parietal Precuneus GM | 7 | -25.76, -61.46, 38.56 | | 4.8 |  |
|  | 143 | Rt Superior Frontal Gyrus GM | 8 | 21.7, 19.7, 43.45 | | 4.74 |  |
|  | 1481 | Rt Superior Temporal Gyrus GM | 38 | 52.88, 0.54, -11.89 | | 4.62 |  |
|  | 626 | Rt Medial Frontal Gyrus GM | 9 | 1.53, 34.91, 32.83 | | 4.45 |  |
|  |  | Lt Frontal Cingulate Gyrus GM | 32 | -3.2, 25.09, 37.23 | | 3.7 |  |
|  | 203 | Lt Frontal Lobe Sub-Gyral GM | 6 | -20.13, 3.96, 52.06 | | 4.25 |  |
|  | 197 | Lt Limbic Cingulate Gyrus GM | 31 | -12.68, -27.73, 39.27 | | 4.11 |  |
|  | 315 | Lt Frontal Postcentral Gyrus GM | 3 | -35.82, -22.19, 41.21 | | 4.03 |  |
|  | 305 | Rt Middle Temporal Gyrus GM | 22 | 54.36, -36.96, 6.2 | | 4.03 |  |
|  |  | Rt Superior Temporal Gyrus GM | 22 | 57.98, -41.23, 11.26 | | 3.48 |  |
|  | 184 | Rt Parietal Lobe Precuneus GM | 19 | 25.22, -70.25, 30.48 | | 3.98 |  |
|  | 211 | Rt Sub-lobar Claustrum GM |  | 30.45, 13.65, 8.79 | | 3.97 |  |
|  | 101 | Rt Inferior Temporal Gyrus GM | 20 | 52.77, -31.31, -13.11 | | 3.77 |  |
|  | 101 | Lt Middle Temporal Gyrus GM | 21 | -54.46, -18.96, -20.06 | | 3.71 |  |
|  | 112 | Rt Frontal Precentral Gyrus GM | 4 | 40.15, -15.54, 37.72 | | 3.69 |  |
|  | 154 | Rt Sub-lobar Insula GM | 13 | 48.73, -21.96, 16.53 | | 3.67 |  |
|  | 237 | Lt Transverse Temporal Gyrus GM | 41 | -45.66, -22.32, 14 | | 3.66 |  |
| **WMV** | 241 | Rt Middle Frontal Gyrus WM | 8 | 30.96, 19.73, 42.7 | | 5.2 |  |
|  | 520 | Rt Parietal Lobe Sub-Gyral WM |  | 39.17, -41.53, 34.34 | | 4.34 |  |
|  | 166 | Rt Superior Temporal Gyrus WM |  | 37.21, 1.06, -16.61 | | 4.14 |  |
|  | 252 | Rt Limbic Cingulate Gyrus WM |  | 14.2, -50.37, 29.47 | | 4.12 |  |
|  | 103 | Rt Frontal Precentral Gyrus WM |  | 29.82, -14.76, 49.33 | | 3.86 |  |
|  | 114 | Lt Frontal Lobe Sub-Gyral WM |  | -20.24, 29.19, -4.11 | | 3.77 |  |
|  | 110 | Rt Frontal Lobe Sub-Gyral WM |  | 13.14, -29.49, 46.75 | | 3.55 |  |
| **gwJTV** | 498 | Rt Sub-lobar Extra-Nuclear |  | 20, -50.43, 10.65 | | 4.38 | ROI |
|  | 154 | Lt Parietal Postcentral Gyrus |  | -37.68, -23.1141.09 | | 4.37 |  |
|  | 1386 | Lt Anterior Cerebellar Lingual |  | -1.87, -46.02-14.53 | | 3.9 |  |
|  |  | Lt Cerebellum Anterior Lobe |  | -7.3, -42.32-23.28 | | 3.84 |  |
|  |  | Rt Anterior Lobe Culmen |  | 4.67, -43.75-18.71 | | 3.79 |  |
|  | 329 | Rt Sub-lobar Extra-Nuclear |  | 16.53, 9.829.99 | | 3.83 |  |
|  |  | Rt Sub-lobar Caudate Body |  | 9.99, 4.7613.91 | | 3.56 |  |
|  | 185 | Right Posterior Cerebellar Tonsil GM |  | 1.09, -54.71-31.52 | | 3.74 |  |

Two-sample t test was performed with a significance level of p = 0.0005 and clusters with at least 100 contiguous voxels. Age was used as a covariate. No significant difference was found in the results of without plaque < with plaque.

The following metrics are used together to interpret the results of voxel-based analyses, providing insights into which areas of the brain are involved in particular tasks or conditions and the strength of these effects. First, the **cluster size** refers to the number of contiguous voxels (3D pixels) that are considered part of a significant cluster. A cluster is a group of voxels that all show a statistically significant effect, such as increased or decreased activity, and are adjacent to one another in the 3D space of the brain image. Second, the **cluster location** indicates the anatomical location of the cluster within the brain. It helps researchers understand which specific brain regions are involved in the observed activity or effect. This is often described in terms of brain regions or structures. Third, the **Talairach coordinates** are a set of three-dimensional coordinates (x, y, z) used to describe the location of brain structures in a standardized space. The Talairach space is based on the brain atlas developed by Jean Talairach and Pierre Tournoux, which allows for consistent location mapping across different individuals' brains. These coordinates help in pinpointing the exact location of clusters in the brain. Finally, the **Z-score** is a statistical measure that describes the number of standard deviations a data point (in this case, the voxel's signal) is from the mean of a set of data. In the context of voxel-based analysis, a higher absolute Z-score indicates a stronger deviation from the mean activity, suggesting a more statistically significant effect. Positive Z-scores typically indicate increased activity, while negative Z-scores indicate decreased activity compared to a baseline or control condition.

*Abbreviation*: GMV, gray matter volume; WMV, white matter volume; gwJTV, gray-white matter junction volume; BA - Brodmann area; Rt, right; Lt, left.

**Supplementary Table S3. Significantly associated areas of results of voxel-based multiple regression analyses between gray-white matter junction tissue volume (gwJTV) and levels of metabolic parameters related to Type 2 diabetes of all participants.**

| **Group analysis** | **Cluster size** | **Cluster location** | **BA** | | **Talairach coordinates** | | **Z score** | | **ROI** |
| --- | --- | --- | --- | --- | --- | --- | --- | --- | --- |
| **AST** | | | | | | | | | |
| **gwJTV (+)** | 1930 | Rt Posterior Inferior Semi-Lunar Lobule | |  | | 17.74, -70.37, -35.42 | | 4.99 |  |
|  |  | Rt Posterior Cerebellar Tonsil | |  | | 29.84, -61.7, -38 | | 3.72 |  |
|  | 577 | Lt Inferior Parietal Lobule | |  | | -37.69, -32.34, 39.31 | | 4.94 |  |
|  | 103 | Rt Frontal Cingulate Gyrus | |  | | 6.97, 10.31, 34.2 | | 4.39 |  |
|  | 224 | Lt Sub-lobar Extra-Nuclear | |  | | -22.24, -14, -4.63 | | 4.34 |  |
|  | 314 | Left Brainstem Midbrain | |  | | -9.33, -18.06, -2.1 | | 4.25 |  |
|  | 107 | Lt Frontal Precentral Gyrus | |  | | -34.11, -16.81, 53.46 | | 4.18 |  |
|  | 105 | Rt Frontal Lobe Sub-Gyral | |  | | 30.8, -19.83, 43.46 | | 4.14 |  |
|  | 834 | Rt Occipital Cuneus | |  | | 8.86, -71.63, 6.65 | | 3.87 |  |
|  |  | Rt Occipital Lobe | |  | | 4.29, -64.82, 4.51 | | 3.84 |  |
|  | 382 | Lt Sub-lobar Extra-Nuclear | |  | | -15.13, -4.28, 20.73 | | 3.86 |  |
|  |  | Lt Sub-lobar Caudate Head | |  | | -8.38, 6.83, 2.98 | | 3.59 |  |
|  | 149 | Rt Occipital Lobe Sub-Gyral | |  | | 25.56, -87.87, -0.91 | | 3.79 |  |
|  | 118 | Rt Sub-lobar Extra-Nuclear | |  | | 15.46, -3.16, 17.75 | | 3.78 |  |
|  | 185 | Rt Frontal Precentral Gyrus | | 6 | | 51.55, -2.42, 18.43 | | 3.76 |  |
| **ApoB** | | | | | | | | | |
| **gwJTV (+)** | 261 | Lt Parietal Lobe Sub-Gyral | | 40 | | -28.61, -42.84, 50.18 | | 3.68 |  |
|  | 125 | Lt Inferior Parietal Lobule | | 7 | | -38.78, -60.98, 43.79 | | 3.61 |  |
| **Cpep** | | | | | | | | | |
| **gwJTV (-)** | 227 | Rt Parietal Postcentral Gyrus | |  | | 34.26, -34.46, 58.35 | | 4.15 |  |
| **Cr** | | | | | | | | | |
| **gwJTV (-)** | 214 | Lt Occipital Cuneus | |  | | -16.46, -84.01, 28.47 | | 3.94 |  |
| **HScrp** | | | | | | | | | |
| **gwJTV (-)** | 263 | Rt Frontal Paracentral Lobule | | 5 | | 4.63, -38.96, 57.42 | | 4.05 |  |
| **HbA1c** | | | | | | | | | |
| **gwJTV (+)** | 224 | Lt Superior Temporal Gyrus | |  | | -37.42, -36.77, 17.28 | | 4.25 |  |
|  | 112 | Rt Frontal Lobe Sub-Gyral | |  | | 31.8, -16.52, 38.38 | | 4 |  |
| **LDL** | | | | | | | | | |
| **gwJTV (+)** | 508 | Rt Parietal Precuneus | |  | | 13.88, -54.59, 53.39 | | 4.97 | ROI |
|  |  | Rt Parietal Lobe Sub-Gyral | | 7 | | 22.22, -50.9, 53.88 | | 3.8 | ROI |
|  |  | Rt Parietal Precuneus | | 7 | | 13.06, -52.87, 45.43 | | 3.76 |  |
| **SBP** | | | | | | | | | |
| **gwJTV (+)** | 456 | Lt Sub-lobar Extra-Nuclear | |  | | -14.02, 5.32, 9.05 | | 4.15 |  |
|  | 171 | Lt Frontal Lobe Sub-Gyral | |  | | -36.66, -25.22, 33.7 | | 3.89 |  |
|  | 130 | Lt Frontal Lobe Sub-Gyral | |  | | -29.64, 28.11, 7.34 | | 3.77 |  |
|  | 158 | Lt Sub-lobar Lentiform Nucleus Lateral Globus Pallidus | |  | | -26.02, -12.73, 1.73 | | 3.67 |  |
| **TC** | | | | | | | | | |
| **gwJTV (+)** | 325 | Rt Parietal Precuneus | | 7 | | 13.01, -55.08, 48.83 | | 4.42 | ROI |
|  | 109 | Lt Frontal Paracentral Lobule | |  | | -11.9, -27, 51.06 | | 4.1 |  |
|  | 307 | Lt Parietal Lobe Sub-Gyral | |  | | -24.89, -42.68, 48.46 | | 3.92 | ROI |
| **TG** | | | | | | | | | |
| **gwJTV (+)** | 362 | Lt Frontal Lobe Sub-Gyral | |  | | -28.77, 35.03, 13.42 | | 4.7 |  |
|  |  | Lt Frontal Lobe Sub-Gyral | |  | | -26.83, 34.79, 6.22 | | 3.86 |  |

Multiple regression was performed with a significance level of p = 0.0005 and clusters with at least 100 contiguous voxels. Age was used as a covariate. Results are shown as positive (＋) and negative (-) association with gwJTV.

*Abbreviation*: gwJTV, gray-white matter junction volume; BA - Brodmann area; Rt, right; Lt, left; ApoB, apolipoprotein B; AST, aspartate aminotransferase; Cpep, C-peptide; Cr, creatinine; HScrp, high-sensitivity C-reactive protein; HbA1c, Glycated haemoglobin; LDL, low-density lipoprotein SBP, systolic blood pressure; TC, total cholesterol; TG, triglyceride.

**Supplementary Table S4. Significantly associated areas of results of voxel-based multiple regression analyses between gray matter volume (GMV) and levels of metabolic parameters related to Type 2 diabetes of all participants.**

| **Group analysis** | **Cluster size** | **Cluster location** | **BA** | | | **Talairach coordinates** | | **Z score** | **ROI** |
| --- | --- | --- | --- | --- | --- | --- | --- | --- | --- |
| **AST** | | | | | | | | | |
| **GMV (+)** | 416 | Rt Middle Temporal Gyrus GM | 22 | | | 52.57, -42.86, -0.7 | | 4.72 |  |
|  | 164 | Lt Occipital Cuneus GM | 18 | | | -9.83, -86.64, 16.62 | | 4.27 |  |
|  | 656 | Lt Middle Temporal Gyrus GM | 21 | | | -52.85, -31.72, -5.03 | | 4.24 |  |
|  | 192 | Lt Anterior Lobe Culmen GM |  | | | -19.54, -46.54, -8.57 | | 4.08 |  |
|  | 211 | Rt Parietal Postcentral Gyrus GM | 3 | | | 31.72, -22.63, 43.21 | | 4.07 |  |
|  | 146 | Lt Inferior Temporal Gyrus GM | 20 | | | -52.85, -50, -10.36 | | 4.04 |  |
|  | 252 | Lt Occipital Lobe Precuneus GM | 31 | | | -24.63, -69.12, 20.73 | | 3.87 |  |
|  | 185 | Lt Occipital Lobe Cuneus GM | 18 | | | -5.16, -76.24, 15.88 | | 3.69 |  |
|  | 131 | Lt Middle Occipital Gyrus GM | 19 | | | -38.34, -69.61, 6.94 | | 3.64 |  |
|  | 169 | Rt Cerebellar Tonsil GM |  | | | 38.11, -61.25, -33.31 | | 3.57 |  |
| **ApoA1** | | | | | | | | | |
| **GMV (+)** | 237 | Rt Posterior Lobe Tuber GM | |  | | | 37.05, -76.85, -27.6 | 4.12 |  |
|  |  | Rt Posterior Lobe Uvula GM | |  | | | 29.61, -79.87, -25.31 | 3.72 |  |
|  | 235 | Lt Frontal Subcallosal Gyrus GM | | 13 | | | -17.4, 14.97, -12.62 | 3.91 |  |
|  | 126 | Lt Middle Frontal Gyrus GM | | 6 | | | -19.17, 20.64, 54.55 | 3.89 |  |
|  | 287 | Rt Parietal Postcentral Gyrus GM | | 3 | | | 22.3, -27.44, 54.3 | 3.83 |  |
|  |  | Rt Frontal Paracentral Lobule GM | | 6 | | | 13.92, -30.54, 57.47 | 3.73 |  |
|  | 112 | Rt Frontal Lobe Precentral Gyrus GM | | 6 | | | 47.43, -5.53, 50.5 | 3.83 |  |
|  | 256 | Lt Frontal Lobe Precentral Gyrus GM | | 4 | | | -45.92, -16.03, 36.21 | 3.76 |  |
|  | 160 | Lt Superior Frontal Gyrus GM | | 8 | | | -7.93, 40.75, 50.34 | 3.73 |  |
|  | 121 | Rt Sub-lobar Insula GM | | 13 | | | 38.03, -2.44, -9.72 | 3.72 |  |
| **BMI** | | | | | | | | | |
| **GMV**  **(-)** | 232 | Lt Inferior Temporal Gyrus GM | | 20 | | | -34.74, -0.46, -36.89 | 4.17 |  |
|  | 548 | Lt Medial Frontal Gyrus GM | | 10 | | | -16.37, 38.6, -13.96 | 4.02 | ROI |
|  | 266 | Rt Inferior Frontal Gyrus GM | | 47 | | | 17.86, 35.63, -13.67 | 4.01 |  |
| **Cpep** | | | | | | | | | |
| **GMV**  **(-)** | 720 | Lt Parietal Postcentral Gyrus GM | | 2 | | | -51.62, -25.34, 45.15 | 4.7 |  |
|  | 440 | Lt Limbic Lobe Uncus GM | | 36 | | | -15.38, -0.24, -30.24 | 4.17 |  |
|  | 424 | Rt Parietal Postcentral Gyrus GM | | 3 | | | 34.25, -34.54, 59.24 | 4.17 |  |
|  | 118 | Lt Inferior Temporal Gyrus GM | | 21 | | | -60.92, -7.84, -18.22 | 4.09 |  |
|  | 231 | Lt Inferior Frontal Gyrus GM | | 46 | | | -48.07, 37.95, 3.46 | 3.89 |  |
|  | 231 | Lt Superior Frontal Gyrus GM | | 9 | | | -4.89, 52.73, 33.51 | 3.89 |  |
|  | 108 | Lt Superior Parietal Lobule GM | | 7 | | | -28.76, -50.32, 59.38 | 3.83 |  |
|  | 265 | Lt Inferior Frontal Gyrus GM | | 11 | | | -12.65, 32.32, -17.2 | 3.76 |  |
|  | 148 | Lt Superior Frontal Gyrus GM | | 8 | | | -4.28, 32.18, 51.39 | 3.75 |  |
|  | 223 | Lt Occipital Lobe Cuneus GM | | 19 | | | -20.22, -86.29, 32.69 | 3.74 |  |
|  | 136 | Rt Medial Frontal Gyrus GM | | 10 | | | 6.45, 60.85, 17.36 | 3.69 |  |
|  | 166 | Lt Inferior Parietal Lobule GM | | 40 | | | -50.71, -41.01, 41.87 | 3.68 |  |
|  | 141 | Rt Superior Temporal Gyrus GM | | 38 | | | 38.29, 18.69, -23.93 | 3.57 |  |
| **HDL** | | | | | | | | | |
| **GMV (+)** | 920 | Rt Medial Frontal Gyrus GM | | 10 | | | 22.39, 43.12, -3.87 | 4.6 |  |
|  | 148 | Rt Limbic Anterior Cingulate GM | | 32 | | | 11.15, 44.93, 6.92 | 4.07 |  |
|  | 335 | Rt Frontal Lobe Sub-Gyral GM | | 4 | | | 16.71, -29.53, 56.71 | 4.05 |  |
|  |  | Rt Frontal Precentral Gyrus GM | | 4 | | | 28.74, -29.6, 56.91 | 3.35 |  |
|  | 1625 | Lt Frontal Subcallosal Gyrus GM | | 47 | | | -21.1, 16.85, -12.5 | 4.02 |  |
|  |  | Lt Inferior Frontal Gyrus GM | | 47 | | | -15.49, 24.53, -14.38 | 3.91 |  |
|  |  | Lt Middle Frontal Gyrus GM | | 11 | | | -19.18, 34.71, -12.58 | 3.73 |  |
|  | 119 | Lt Superior Frontal Gyrus GM | | 9 | | | -9.57, 53.34, 37.09 | 3.95 |  |
|  | 516 | Rt Parietal Postcentral Gyrus GM | | 5 | | | 6.35, -40.86, 67.18 | 3.87 |  |
|  |  | Rt Frontal Paracentral Lobule GM | | 5 | | | 10.15, -43.82, 58.85 | 3.69 |  |
|  | 144 | Rt Middle Temporal Gyrus GM | | 21 | | | 59.41, -4.56, -17.67 | 3.74 |  |
|  | 106 | Rt Frontal Precentral Gyrus GM | | 6 | | | 47.46, -5.27, 47.82 | 3.71 |  |
|  | 322 | Rt Medial Frontal Gyrus GM | | 10 | | | 14.99, 60.51, 1.25 | 3.7 |  |
|  | 111 | Rt Parietal Lobe Precuneus GM | | 7 | | | 9.26, -55.41, 52.33 | 3.68 |  |
|  | 140 | Rt Sub-lobar Extra-Nuclear GM | | | 13 | | 38.04, -1.42, -10.53 | 3.66 |  |
| **HOMAIR** | | | | | | | | | |
| **GMV**  **(-)** | 1613 | Lt Medial Frontal Gyrus GM | | | 25 | | -14.6, 15.13, -14.36 | 4.5 | ROI |
|  | 546 | Rt Medial Frontal Gyrus GM | | | 25 | | 13.16, 14.98, -13.9 | 4.24 |  |
|  | 132 | Rt Middle Temporal Gyrus GM | | | 39 | | 46.69, -67.19, 17.62 | 3.86 |  |
| **LDL** | | | | | | | | | |
| **GMV (+)** | 172 | Lt Frontal Precentral Gyrus GM | | | 6 | | -31.37, -16.16, 56.27 | 4.25 |  |
|  | 270 | Lt Frontal Precentral Gyrus GM | | | 9 | | -44.83, 18.96, 34.14 | 4.24 |  |
|  | 128 | Rt Superior Frontal Gyrus GM | | | 6 | | 4.83, 13.64, 57.9 | 4.02 |  |
|  | 257 | Lt Frontal Subcallosal Gyrus GM | | | 47 | | -16.48, 18.6, -11.36 | 3.84 |  |
|  | 241 | Rt Superior Frontal Gyrus GM | | | 11 | | 12.37, 50.74, -14.13 | 3.78 |  |
|  |  | Rt Medial Frontal Gyrus GM | | | 10 | | 16.92, 51.97, -7.63 | 3.43 |  |
| **SBP** | | | | | | | | | |
| **GMV**  **(-)** | 293 | Lt Superior Frontal Gyrus GM | | | 6 | | -5.4, 17.92, 62.64 | 4.21 |  |
|  |  | Lt Superior Frontal Gyrus GM | | | 6 | | -12.81, 17.03, 62.43 | 4.17 |  |
|  | 243 | Rt Frontal Precentral Gyrus GM | | | 6 | | 55.95, -2.32, 36.53 | 4.01 |  |
|  | 114 | Rt Middle Frontal Gyrus GM | | | 9 | | 48.62, 10.18, 33.99 | 4 |  |
| **TC** | | | | | | | | | |
| **GMV (+)** | 218 | Lt Frontal Precentral Gyrus GM | | | 4 | | -30.4, -15.9, 53.61 | 4.58 |  |
| **insulinA** | | | | | | | | | |
| **GMV**  **(-)** | 995 | Lt Inferior Frontal Gyrus GM | | | 47 | | -21.99, 21.69, -13.86 | 4.14 |  |
|  |  | Lt Medial Frontal Gyrus GM | | | 25 | | -12.74, 18.84, -13.97 | 3.84 |  |
|  | 157 | Rt Medial Frontal Gyrus GM | | | 25 | | 13.18, 17.86, -14.53 | 3.61 |  |

Multiple regression was performed with a significance level of p = 0.0005 and clusters with at least 100 contiguous voxels. Age was used as a covariate. Results are shown as positive (＋) and negative (-) association with GMV.

*Abbreviation*: GMV, gray matter volume; BA - Brodmann area; Rt, right; Lt, left; ApoA1, apolipoprotein A1; AST, aspartate aminotransferase; BMI, body mass index; Cpep, C-peptide; HDL, high-density lipoprotein; HOMAIR, Homeostasis model assessment of insulin resistance; LDL, low-density lipoprotein; SBP, systolic blood pressure; TC, total cholesterol;

**Supplementary Table S5. Significantly associated areas of results of voxel-based multiple regression analyses between white matter volume (WMV) and levels of metabolic parameters related to Type 2 diabetes of all participants.**

| **Group analysis** | **Cluster size** | **Cluster location** | **BA** | | **Talairach coordinates** | **Z score** | | **ROI** |
| --- | --- | --- | --- | --- | --- | --- | --- | --- |
| **AST** | | | | | | | | |
| **WMV (+)** | 1771 | Lt Sub-lobar temporal WM |  | | -36.93, -16.69, -15.05 | 4.88 | |  |
|  | 248 | Lt Inferior Parietal Lobule WM |  | | -36.77, -33.28, 39.24 | 4.59 | |  |
|  | 152 | Lt Limbic Cingulate WM | 24 | | -11.78, -5.05, 47.74 | 3.75 | |  |
|  | 460 | Rt Temporal Lobe Sub-Gyral WM |  | | 37.12, -14.2, -14.46 | 3.68 | |  |
|  | 618 | Rt Anterior Lobe Culmen WM |  | | 15.81, -46.26, -22.36 | 3.63 | |  |
|  |  | Rt Posterior Cerebellar Tonsil WM |  | | 23.31, -60.24, -33.47 | 3.53 | |  |
| **ApoA1** | | | | | | | | |
| **WMV (+)** | 402 | Rt Frontal Lobe Sub-Gyral WM | |  | 13.95, -20.29, 58.44 | | 4.47 |  |
|  | 149 | Lt Medial Frontal Gyrus WM | |  | -12.81, -14.04, 53.18 | | 3.83 |  |
| **Cpep** | | | | | | | | |
| **WMV (-)** | 439 | Rt Frontal Precentral WM | |  | 27.89, -22.64, 53.05 | | 4.48 |  |
|  | 126 | Lt Occipital Lingual WM | |  | -16.96, -87.31, -5.18 | | 3.84 |  |
| **Cr** | | | | | | | | |
| **WMV (-)** | 989 | Left Brainstem Midbrain | |  | -13.79, -18.59, -15.73 | | 5.04 | ROI |
|  | 251 | Rt Medial Frontal WM | |  | 14, -16.22, 55.23 | | 4.14 |  |
|  | 607 | Lt Sub-lobar Extra-Nuclear WM | |  | -25.83, 25.23, -1.88 | | 4.13 |  |
|  | 189 | Rt Superior Temporal WM | |  | 35.45, 4.47, -22.63 | | 4.1 |  |
|  | 436 | Lt Occipital Cuneus WM | |  | -17.33, -81.8, 25.06 | | 3.93 |  |
|  |  | Lt Occipital Precuneus WM | | 31 | -20.02, -72.96, 21.35 | | 3.71 |  |
| **DBP** | | | | | | | | |
| **WMV (-)** | 272 | Rt Frontal Postcentral Gyrus WM | |  | 42.04, -21.64, 32.67 | | 4.16 |  |
| **HDL** | | | | | | | | |
| **WMV (+)** | 621 | Rt Temporal Lobe Sub-Gyral WM | |  | 24.41, -63.03, 23.94 | | 4.39 |  |
|  | 174 | Lt Medial Frontal WM | |  | -7.26, -14.16, 54.16 | | 4.2 |  |
|  | 239 | Rt Frontal Lobe Sub-Gyral WM | |  | 14.87, -19.36, 58.55 | | 4.06 |  |

Multiple regression was performed with a significance level of p = 0.0005 and clusters with at least 100 contiguous voxels. Age was used as a covariate. Results are shown as positive (＋) and negative (-) association with WMV.

*Abbreviation*: WMV, white matter volume; BA - Brodmann area; Rt, right; Lt, left; ApoA1, apolipoprotein A1; AST, aspartate aminotransferase; Cr, creatinine; Cpep, C-peptide; DBP, diastolic blood pressure; HDL, high-density lipoprotein.

**Supplementary Table S6. Results of multiple regression analysis of brain tissue volumes with blood biomarkers in specific brain areas in the participants without plaques**

| **ROIs** | **BTV** | **Initial Model Inputs (Step1)** | **Final Model Inputs**  **(Step2)** | **Significant Result** |
| --- | --- | --- | --- | --- |
| **Cluster 1**  Rt Medial Frontal Gyrus | GMV | Age, HbA1c, ApoA1, HDL, HOMAIR, insulinA, BMI, Mdur | BMI, DMdur | ***BMI*** ***(β= -0.011, p=0.005)***  ***DMdur*** ***(β= -0.005, p= 0.007)*** |
|  | WMV | Age, HbA1c |  | No significance |
|  | gwJTV | Age, HbA1c, ALT | ALT | No significance |
| **Cluster 2**  Lt Medial Frontal Gyrus | GMV | age, HbA1c, ApoA1, HDL, BMI | HDL, BMI | HDL (β= 0.002, p=0.050)  ***BMI (β= -0.010, p= 0.001)*** |
|  | WMV | age, HbA1c, ALT, ApoA1 | ALT, ApoA1 | No significance |
|  | gwJTV | Age, HbA1c, ApoA1, HDL, BMI | HDL | HDL (β= 0.001, p= 0.038) |
| **Cluster 3**  Rt Parietal Lobe | GMV | Age, HbA1c |  | No significance |
|  | WMV | Age, HbA1c, LDL | LDL | No significance |
|  | gwJTV | Age, HbA1c, ApoA1, HDL, HOMAIR, insulinA, BMI | BMI | ***BMI*** ***(β= -0.011, p= 0.003)*** |
| **Cluster 4**  Lt Parietal Lobe | GMV | Age, HbA1c, SBP | SBP | No significance |
|  | WMV | Age, HbA1c |  | No significance |
|  | gwJTV | Age, HbA1c, ApoB, BA1ratio, LDL, TC | Age | Age (β= 0.003, p= 0.035) |
| **Cluster 5**  Left Brainstem Midbrain | GMV | Age, HbA1c, HOMAIR, insulin A |  | No significance |
|  | WMV | Age, HbA1c, Cr, HDL | Cr | ***Cr (β= -0.203, p= 0.0001)*** |
|  | gwJTV | Age, HbA1c |  | No significance |
| **Cluster 6**  Lt Middle Temporal Gyrus | GMV | Age, HbA1c, logLpa | Age | No significance |
|  | WMV | Age, HbA1c | Age | Age (β= -0.001, p= 0.033) |
|  | gwJTV | Age, HbA1c | Age | Age (β= -0.001, p= 0.044) |
| **Cluster 7**  Rt Sub-lobar Extra-Nuclear | GMV | Age, HbA1c, GFR1 |  | No significance |
|  | WMV | Age, HbA1c |  | No significance |
|  | gwJTV | Age, HbA1c |  | No significance |
| **Hippocampus** | GMV | Age, HbA1c, HOMAIR | Age, HOMAIR | No significance |
|  | WMV | Age, HbA1c, AST, HOMAIR | Age, AST, HOMAIR | No significance |
|  | gwJTV | Age, HbA1c, HOMAIR | Age | ***Age*** ***(β= -0.002, p= 0.0002)*** |
| **Middle temporal gyrus** | GMV | Age, HbA1c, AST | Age | Age (β= -0.002, p= 0.026) |
|  | WMV | Age, HbA1c |  | No significance |
|  | gwJTV | Age, HbA1c |  | No significance |
| **Superior frontal gyrus** | GMV | Age, HbA1c, ApoA1, HDL | HbA1c, ApoA1 | HbA1c (β= 0.013, p= 0.025)  ***ApoA1 (β= 0.001, p= 0.005)*** |
|  | WMV | Age, HbA1c |  | No significance |
|  | gwJTV | Age, HbA1c, ApoA1, HDL | Age, HbA1c, HDL | ***HDL (β= 0.001, p= 0.009)*** |
| **Thalamus** | GMV | Age, HbA1c |  | No significance |
|  | WMV | Age, HbA1c | Age | No significance |
|  | gwJTV | Age, HbA1c, AST | AST | No significance |

Results of stepwise multiple regression analysis for the association between brain tissue volumes and blood biomarkers are listed as correlation coefficient (β) and p-value. The parameters input in the model were determined by results of partial correlation analysis. The initial model is ROI tissue volume = β1*age+ β2*HbA1c+...+ βn* blood biomarkers+ error. The variables were included in the model with p< 0.05 and excluded in the model with p> 0.1. The cluster ROI areas are summarized in **Supplementary Table S1**. *Italic* and **bold** characters indicate a significant correlation between MRI measures and biomarkers with p=**0.016** (p=0.05/3, which are GMV, WMV, and gwJTV).

*Abbreviation*: BTV, brain tissue volume; GMV, gray matter volume; WMV, white matter volume; gwJTV, gray-white matter junction tissue volume; ALT, Alanine aminotransferase; ApoA1, apolipoprotein A1; ApoB, apolipoprotein B; AST, aspartate aminotransferase; BA1ratio, apolipoprotein A1/B ratio; BMI, body mass index; Cr, creatinine; DMdur, duration of diabetes (years); HbA1c, Glycated hemoglobin; HDL, high-density lipoprotein; HOMAIR, Homeostasis model assessment of insulin resistance; HScrp, high-sensitivity C-reactive protein; LDL, low-density lipoprotein; SBP, systolic blood pressure; TC, total cholesterol; TG, triglyceride.

**Supplementary Table S7. Results of multiple regression analysis of brain tissue volumes with blood biomarkers in specific brain areas in the participants with plaques**

| **ROIs** | **BTV** | **Initial Model Inputs**  **(Step1)** | **Final Model Inputs (Step2)** | **Significant Result** |
| --- | --- | --- | --- | --- |
| **Cluster 1**  Rt Medial Frontal Gyrus | GMV | Age, HbA1c, ApoA1, HDL, HOMAIR, insulinA, BMI, Mdur | HOMAIR | ***HOMAIR (β= -0.002, p= 0.0001)*** |
|  | WMV | Age, HbA1c |  | No significance |
|  | gwJTV | Age, HbA1c, ALT | ALT | No significance |
| **Cluster 2**  Lt Medial Frontal Gyrus | GMV | age, HbA1c, ApoA1, HDL, BMI | BMI | No significance |
|  | WMV | age, HbA1c, ALT, ApoA1 | ALT, ApoA1 | ALT (β= 0.00008, p= 0.039)  ApoA1 (β= 0.00008, p= 0.021) |
|  | gwJTV | Age, HbA1c, ApoA1, HDL, BMI | BMI | No significance |
| **Cluster 3**  Rt Parietal Lobe | GMV | Age, HbA1c |  | No significance |
|  | WMV | Age, HbA1c, LDL | LDL | No significance |
|  | gwJTV | Age, HbA1c, ApoA1, HDL, HOMAIR, insulinA, BMI | HDL | HDL (β= 0.002, p= 0.018) |
| **Cluster 4**  Lt Parietal Lobe | GMV | Age, HbA1c, SBP | SBP | No significance |
|  | WMV | Age, HbA1c |  | No significance |
|  | gwJTV | Age, HbA1c, ApoB, BA1ratio, LDL, TC | TC | ***TC (β= 0.002, p= 0.001)*** |
| **Cluster 5**  Left Brainstem Midbrain | GMV | Age, HbA1c, HOMAIR, insulin A |  | No significance |
|  | WMV | Age, HbA1c, Cr, HDL | Cr | ***Cr (β= -0.124, p=0.003)*** |
|  | gwJTV | Age, HbA1c | Age | ***Age (β= -0.004, p= 0.001)*** |
| **Cluster 6**  Lt Middle Temporal Gyrus | GMV | Age, HbA1c, logLpa | logLpa | ***logLpa (β= 0.040, p= 0.003)*** |
|  | WMV | Age, HbA1c |  | No significance |
|  | gwJTV | Age, HbA1c |  | No significance |
| **Cluster 7**  Rt Sub-lobar Extra-Nuclear | GMV | Age, HbA1c, GFR1 | GFR1 | GFR1 (β= -0.0009, p= 0.041) |
|  | WMV | Age, HbA1c |  | No significance |
|  | gwJTV | Age, HbA1c |  | No significance |
| **Hippocampus** | GMV | Age, HbA1c, HOMAIR | Age, HOMAIR | No significance |
|  | WMV | Age, HbA1c, AST, HOMAIR | Age | Age (β= -0.0005, p= 0.049) |
|  | gwJTV | Age, HbA1c, HOMAIR | Age, HOMAIR | No significance |
| **Middle temporal gyrus** | GMV | Age, HbA1c, AST | Age, AST | No significance |
|  | WMV | Age, HbA1c | Age | Age (β= -0.0007, p= 0.047) |
|  | gwJTV | Age, HbA1c |  | No significance |
| **Superior frontal gyrus** | GMV | Age, HbA1c, ApoA1, HDL | HbA1c, AST | No significance |
|  | WMV | Age, HbA1c |  | No significance |
|  | gwJTV | Age, HbA1c, ApoA1, HDL | ApoA1 | No significance |
| **Thalamus** | GMV | Age, HbA1c |  | No significance |
|  | WMV | Age, HbA1c | Age | Age (β= -0.0015, p= 0.027) |
|  | gwJTV | Age, HbA1c, AST | AST | No significance |

Results of stepwise multiple regression analysis for the association between brain tissue volumes and blood biomarkers are listed as correlation coefficient (β) and p-value. The parameters input in the model were determined by results of partial correlation analysis. The initial model is ROI tissue volume = β1*age+ β2*HbA1c+...+ βn* blood biomarkers+ error. The variables were included in the model with p< 0.05 and excluded in the model with p> 0.1. The cluster ROI areas are summarized in **Supplementary Table S1**. *Italic* and **bold** characters indicate a significant correlation between MRI measures and biomarkers with p=**0.016** (p=0.05/3, which are GMV, WMV, and gwJTV).

*Abbreviation*: BTV, brain tissue volume GMV, gray matter volume; WMV, white matter volume; gwJTV, gray-white matter junction tissue volume; ALT, Alanine aminotransferase; ApoA1, apolipoprotein A1; ApoB, apolipoprotein B; AST, aspartate aminotransferase; BA1ratio, apolipoprotein A1/B ratio; BMI, body mass index; Cr, creatinine; DMdur, duration of diabetes (years); HbA1c, Glycated hemoglobin; HDL, high-density lipoprotein; HOMAIR, Homeostasis model assessment of insulin resistance; HScrp, high-sensitivity C-reactive protein; LDL, low-density lipoprotein; SBP, systolic blood pressure; TC, total cholesterol; TG, triglyceride.

**Supplementary Table S8. Results of group comparison of three brain tissue volumes between participants with and without plaque in each brain area, excluding 15 participants with MMSE < 26.**

| **ROIs** | **Tissue volume** | **Without plague** | **With plague** | **Statistics (p)** |
| --- | --- | --- | --- | --- |
| **Cluster 1**  Rt Medial Frontal Gyrus | GMV | 0.398±0.067 | 0.373±0.086 | p= 0.244 |
|  | WMV | 0.012±0.007 | 0.010±0.006 | p= 0.180 |
|  | gwJTV | 0.141±0.030 | 0.137±0.043 | p= 0.677 |
| **Cluster 2**  Lt Medial Frontal Gyrus | GMV | 0.156±0.056 | 0.151±0.059 | p= 0.733 |
|  | WMV | 0.004±0.005 | 0.003±0.004 | p= 0.299 |
|  | gwJTV | 0.062±0.031 | 0.056±0.027 | p= 0.499 |
| **Cluster 3**  Rt Parietal Lobe | GMV | 0.164±0.046 | 0.158±0.057 | p= 0.674 |
|  | WMV | 0.258±0.070 | 0.260±0.112 | p= 0.933 |
|  | gwJTV | 0.182±0.063 | 0.176±0.058 | p= 0.710 |
| **Cluster 4**  Lt Parietal Lobe | GMV | 0.184±0.054 | 0.162±0.052 | p= 0.127 |
|  | WMV | 0.240±0.095 | 0.259±0.092 | p= 0.448 |
|  | gwJTV | 0.252±0.055 | 0.282±0.094 | p= 0.164 |
| **Cluster 5**  Left Brainstem Midbrain | GMV | 0.026±0.009 | 0.024±0.009 | p= 0.460 |
|  | WMV | 0.378±0.062 | 0.381±0.055 | p= 0.869 |
|  | gwJTV | 0.162±0.047 | 0.160±0.052 | p= 0.872 |
| **Cluster 6**  Lt Middle Temporal Gyrus | GMV | 0.386±0.067 | 0.314±0.051 | ***P= 0.0001*** |
|  | WMV | 0.028±0.016 | 0.044±0.035 | p= 0.037 |
|  | gwJTV | 0.143±0.030 | 0.127±0.031 | p= 0.051 |
| **Cluster 7**  Rt Sub-lobar Extra-Nuclear | GMV | 0.103±0.063 | 0.123±0.081 | p= 0.316 |
|  | WMV | 0.365±0.095 | 0.348±0.090 | p= 0.517 |
|  | gwJTV | 0.416±0.099 | 0.330±0.103 | ***p= 0.0027*** |
| **Hippocampus** | GMV | 0.362±0.049 | 0.348±0.055 | p= 0.334 |
|  | WMV | 0.060±0.010 | 0.057±0.011 | p= 0.360 |
|  | gwJTV | 0.127±0.024 | 0.113±0.026 | p= 0.049 |
| **Middle temporal gyrus** | GMV | 0.293±0.035 | 0.280±0.031 | p= 0.153 |
|  | WMV | 0.089±0.011 | 0.091±0.015 | p= 0.495 |
|  | gwJTV | 0.120±0.016 | 0.117±0.012 | p= 0.464 |
| **Superior frontal gyrus** | GMV | 0.194±0.031 | 0.193±0.028 | p= 0.912 |
|  | WMV | 0.053±0.012 | 0.054±0.011 | p= 0.744 |
|  | gwJTV | 0.097±0.018 | 0.098±0.014 | p= 0.769 |
| **Thalamus** | GMV | 0.250±0.040 | 0.242±0.050 | p= 0.498 |
|  | WMV | 0.207±0.032 | 0.202±0.025 | p= 0.568 |
|  | gwJTV | 0.453±0.070 | 0.437±0.065 | p= 0.409 |

Data of three brain tissue volumes for each group are presented as mean±standard deviation. Results of the group comparison of three brain tissue volumes between participants without and with plaque are listed as p-value. *Italic* and **bold** characters in each column indicate a statistical significance.

*Italic* and **bold** characters indicate a significant difference of MRI measures between patients with and without plaques. The p-value of **0.016** (p=0.05/3, which are GMV, WMV, and gwJTV) was used to consider statistical significance.

*Abbreviation*: GMV, gray matter volume; WMV, white matter volume; gwJTV, gray-white matter junction tissue volume; MMSE, Mini-Mental Status Examination

**1.2 Supplementary Figures**


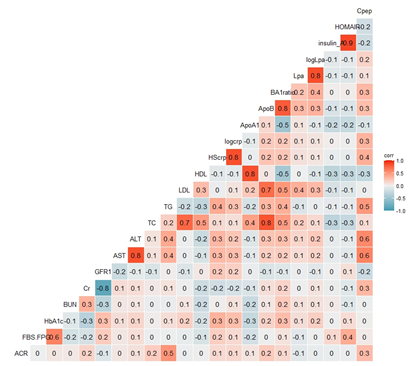


**Supplementary Figure S1. Results of the heatmap analysis between metabolic parameters.**

The warm color indicates the positive correlation between metabolic parameters, but the cool color indicates the negative correlation between them.
